# Conserved CDC20 Cell Cycle Functions Are Carried out by Two of the Five Isoforms in *Arabidopsis thaliana*

**DOI:** 10.1371/journal.pone.0020618

**Published:** 2011-06-08

**Authors:** Zoltán Kevei, Mikhail Baloban, Olivier Da Ines, Hilda Tiricz, Alexandra Kroll, Krzysztof Regulski, Peter Mergaert, Eva Kondorosi

**Affiliations:** 1 Institut des Sciences du Végétal, Centre National de la Recherche Scientifique, Unité Propre de Recherche 2355, Gif-sur-Yvette, France; 2 Institute for Plant Genomics, Human Biotechnology and Bioenergy, Bay Zoltan Foundation for Applied Research, Szeged, Hungary; Instituto de Biología Molecular y Celular de Plantas, Spain

## Abstract

**Background:**

The CDC20 and Cdh1/CCS52 proteins are substrate determinants and activators of the Anaphase Promoting Complex/Cyclosome (APC/C) E3 ubiquitin ligase and as such they control the mitotic cell cycle by targeting the degradation of various cell cycle regulators. In yeasts and animals the main CDC20 function is the destruction of securin and mitotic cyclins. Plants have multiple *CDC20* gene copies whose functions have not been explored yet. In *Arabidopsis thaliana* there are five CDC20 isoforms and here we aimed at defining their contribution to cell cycle regulation, substrate selectivity and plant development.

**Methodology/Principal Findings:**

Studying the gene structure and phylogeny of plant CDC20s, the expression of the five *AtCDC20* gene copies and their interactions with the APC/C subunit APC10, the CCS52 proteins, components of the mitotic checkpoint complex (MCC) and mitotic cyclin substrates, conserved CDC20 functions could be assigned for AtCDC20.1 and AtCDC20.2. The other three intron-less genes were silent and specific for Arabidopsis. We show that AtCDC20.1 and AtCDC20.2 are components of the MCC and interact with mitotic cyclins with unexpected specificity. *AtCDC20.1* and *AtCDC20.2* are expressed in meristems, organ primordia and *AtCDC20.1* also in pollen grains and developing seeds. Knocking down both genes simultaneously by RNAi resulted in severe delay in plant development and male sterility. In these lines, the meristem size was reduced while the cell size and ploidy levels were unaffected indicating that the lower cell number and likely slowdown of the cell cycle are the cause of reduced plant growth.

**Conclusions/Significance:**

The intron-containing *CDC20* gene copies provide conserved and redundant functions for cell cycle progression in plants and are required for meristem maintenance, plant growth and male gametophyte formation. The Arabidopsis-specific intron-less genes are possibly “retrogenes” and have hitherto undefined functions or are pseudogenes.

## Introduction

Consecutive and repeated action of ubiquitin activating (E1), ubiquitin conjugating (E2) and ubiquitin ligase (E3) enzymes leads to polyubiquitination and consequently to degradation of target proteins by the 26S proteasome. Irreversible, spatially and temporally controlled elimination of proteins by this pathway regulates many of the cellular processes. The specificity of the pathway, namely the substrate selection, largely depends on the E3 enzymes. The Anaphase Promoting Complex/Cyclosome (APC/C), a conserved multi-subunit E3 ubiquitin ligase is an essential regulator of the eukaryotic cell cycle [1]. The APC/C complex is composed of at least 11 different core subunits, while the activity and substrate specificity of the APC/C are predominantly determined by two classes of activator proteins: CDC20 and CDH1, the latter known in plants as CCS52 [2]. CDC20 and CDH1 are related proteins, both containing seven WD40 repeats that form a β-propeller structure and represent the major sites for protein interactions. CDC20 and CDH1 interact on the one hand with the APC/C and on the other hand with specific APC/C substrates. Both proteins have in common a C-box motif at the N-terminus and C-terminal IR residues that are required for their binding to the APC/C core. Each of them targets the degradation of proteins containing the loosely defined RxxLxxxN/Q destruction box (D-box) sequence [3]. This sequence was first found in mitotic cyclins that bind to the RLV cyclin-binding motif, conserved in the last WD40 repeat of both the CDC20 and CDH1 proteins. The substrate range of CDH1 is, however, not restricted to D-box proteins as it interacts with a wider range of proteins containing the KEN box or other degradation motifs [4].

APC/C^CDC20^ and APC/C^CDH1^ act one after the other in the cell cycle resulting in controlled temporal degradation of various mitotic regulators ensuring the correct order of the successive cell cycle events [5], [6], [7]. The activity of CDC20 and CDH1 is regulated at multiple levels including transcriptional control, posttranslational modifications (phosphorylation), subcellular localization, protein stability and protein interactions.

In yeast and animal systems expression of *CDC20* is induced at S/G2/M and precedes that of *CDH1* in the cell cycle [7]. CDC20 binds to the APC/C in early mitosis once the core APC/C subunits became phosphorylated. After nuclear envelope breakdown, APC/C^CDC20^ targets CYCLIN A and other substrates for degradation in prometaphase [8]. Later, its activity is temporarily restrained by the “spindle assembly checkpoint” (SAC), which is a surveillance mechanism sensing unattached chromosomes and delaying anaphase by inhibiting APC/C^CDC20^ activity until chromosomes are properly attached and bi-oriented at the metaphase plate [9]. When SAC is activated, the spindle checkpoint proteins (e.g. MAD1, MAD2, BUBR1/MAD3, BUB1) are recruited to the unattached kinetochore. The MAD2, BUBR1/MAD3 and BUB3 proteins, interacting with either free or APC/C bound CDC20, form the mitotic checkpoint complex (MCC). Sequestering CDC20 or APC/C^CDC20^ into the MCC inhibits the APC/C activity. Nevertheless, recent studies indicate that the crucial step in spindle checkpoint arrest is actually not the locked state of CDC20 or APC/C^CDC20^ blocking the access to substrates but the ubiquitination and constant degradation of CDC20 by itself that is triggered by its interaction with MAD2 and BUBR1 [10]. In addition, phosphorylation of CDC20 by BUB1 kinase inhibits also APC/C^CDC20^ catalytically.

When the chromatids are correctly captured by the spindle microtubules and the chromosomes have become bi-oriented on the metaphase plate, the SAC is turned off and APC/C^CDC20^ is released from the inhibitory MCC and becomes active. APC/C^CDC20^ initiates anaphase by degradation of the separase inhibitor securin and cyclin B, leading to the activation of separase enzyme and inhibition of mitotic CDK1 kinase activity that has kept both separase and CDH1 inactive by phosphorylation. Unphosphorylated, securin-free separase cleaves the cohesion protein complex to liberate sister chromatids at anaphase onset. Similarly, being unphosphorylated, CDH1 becomes the activator of the APC/C which then mediates the degradation of CDC20 and regulation of the cell cycle events from mitosis exit to S phase. Degradation of CDC20 is dependent on the D-box in yeasts [11] and on the KEN-box in vertebrates [12]. In mammalian oocytes and embryos, contribution of a further motif (the CRY-box) was also reported in the APC/C^CDH1^ dependent CDC20 degradation [13].

Until recently, postmitotic functions of the APC/C were attributed to APC/C^CDH1^ which was shown to regulate neuronal development [14] or to promote cell cycle exit and endoreduplication in the salivary glands of insects [15]. A recent study demonstrated, however, also a role for APC/C^CDC20^ in neuronal development [16]. In addition, an APC/C independent function has also been reported for CDC20 in budding yeast, promoting spindle elongation and chromosome segregation under replication stress in a DNA damage checkpoint mutant [17].

CDC20 and CDH1/CCS52 are also conserved in the plant kingdom. Unlike other eukaryotes, two types of the CDH1 protein have evolved in plants, CCS52A and CCS52B. They were identified in *Medicago* species; in the cultivated alfalfa and the model legume *Medicago truncatula* where CCS52A proved to be the ortholog of the fission yeast protein and CCS52B to be plant-specific [18]. In *Medicago*, CCS52A controls mitotic exit, cell cycle switch to endoreduplication cycles, resulting in genome doublings and cell differentiation [2], [19]. In *A. thaliana*, the *AtCCS52A* gene is duplicated and the isoforms share the *Medicago* CCS52A functions, mostly on a complementary manner and differing predominantly in their expression pattern. Both proteins control meristem size and maintenance in Arabidopsis roots. AtCCS52A1 stimulates endoreduplication and mitotic exit, delineating the border between the meristem and the elongation zone while AtCCS52A2 controls the identity of the quiescent center cells and stem cell maintenance [20]. AtCCS52A1 was also found to stimulate endoreduplication in trichomes [21].

In spite of the key importance of CDC20 in cell cycle control and cell proliferation, CDC20s have not been characterized yet from plants. In most plant genomes, more than one gene codes for CDC20. In Arabidopsis six *CDC20* genes have been predicted [22] and a recent work revealed the expression for two of the six *AtCDC20s; AtCDC20*.1 and *AtCDC20*.2 during leaf development [23]. However, the significance of multiple *CDC20* gene copies and their specific role in the cell cycle and plant development has not been explored yet.

Our present study aimed at understanding the functionality of the Arabidopsis CDC20 isoforms. Analyzing the structural features of AtCDC20s revealed the existence of five isoforms. We show that AtCDC20.1 and AtCDC20.2 are highly redundant mitotic cell cycle regulators that are expressed in tissues with high cell division activity and are required for normal plant development. On the contrary, *AtCDC20.3*, *AtCDC20.4* and *AtCDC20.5* might be retrogenes which have lost their function as canonical CDC20 genes.

## Results

### Five *CDC20* genes in *Arabidopsis thaliana*

**Figure 1:**
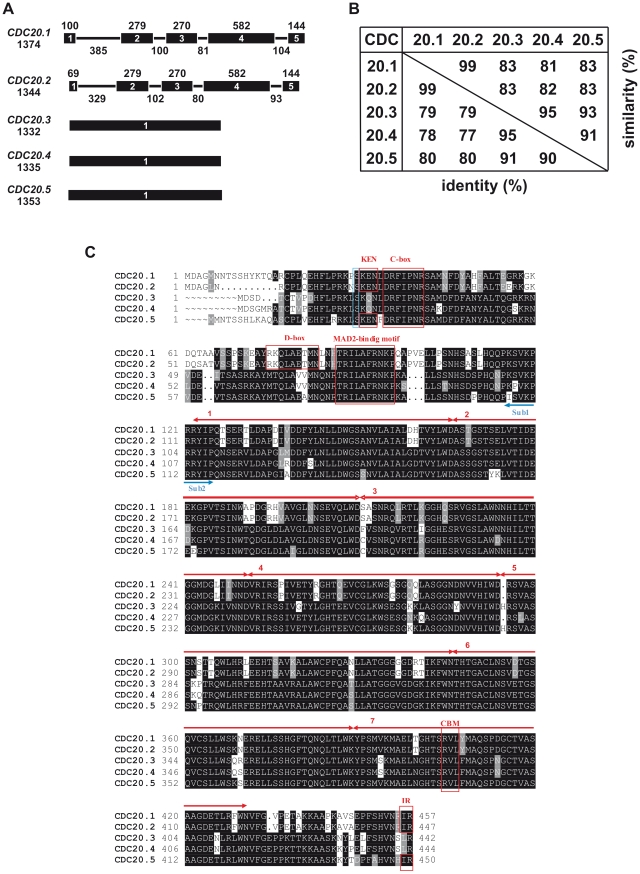
Gene structures and sequence comparisons of *AtCDC20s*. (**A**) Exon-intron sizes in base pairs and organization of *AtCDC20s*. (**B**) Identity and similarity (%) of the different AtCDC20s proteins in pair-wise comparisons. (**C**) Box shade alignment of the AtCDC20 proteins. The characteristic CDC20 motifs (KEN, C-box, D-box, MAD2-binding motif, CBM, IR) are marked with red squares and the seven WD40 repeats with red arrows. Blue arrows mark the AtCDC20 subdomains (sub1 and sub2), used in Y2H assays. Potential BUB1 phosphorylation site at the conserved N-terminal serine residue is circumscribed in blue.

In the Arabidopsis genome six *AtCDC20* genes were predicted by Capron et al. [22]. However, as the hypothetical gene product of At5g27945 was only homologous to the WD40 repeats of CDC20s and lacking the characteristic N-terminal CDC20 structural motifs, we did not consider it as CDC20 and thus studied the other five Arabidopsis AtCDC20 isoforms (Figure 1A). *AtCDC20.1* (At4g33270) and *AtCDC20.2* (At4g33260) genes are located in a 6 kb region on chromosome 4 in the same orientation and separated by a 1 kb of intergenic region. The other three genes, *AtCDC20.3* (At5g27080), *AtCDC20.4* (At5g26900) and *AtCDC20.5* (At5g27570) are clustered on chromosome 5 in a region of less than 300 kb. The gene structure of *AtCDC20.1* and *AtCDC20.2* was similar, each possessing 5 exons separated by 4 introns; in contrast, the *AtCDC20.3*, *AtCDC20.4* and *AtCDC20.5* genes have no introns (Figure 1A). The AtCDC20.1 and AtCDC20.2 proteins are 99% identical, but sharing only 77–80% identity with AtCDC20.3, AtCDC20.4 and AtCDC20.5 that are 90–95% identical amongst each other (Figure 1B).

We were intrigued by the difference in gene organization. To find out whether it is a general feature of plants to have intron-containing and intron-less *CDC20* genes we analyzed the *CDC20* genes and their organization in *Arabidopsis lyrata*, *Vitis vinifera*, *Populus trichocarpa*, *Carica papaya*, *Glycine max*, *Sorghum bicolor*, *Oryza sativa* and *Zea mays*. According to the PLAZA database [24], these plants have between one and six *CDC20* gene copies in their genomes (Figure S1). A phylogenetic analysis of the encoded proteins shows that genes of the same species preferentially cluster together (Figure S1 and Figure S2), indicating that most duplications are recent and occurring in a species- or genus-specific manner. The majority of the identified plant *CDC20* genes have the 5 exon structure as found in *AtCDC20.1* and *AtCDC20.2* with strictly conserved intron-exon boundaries (Figure S2 and Figure S3). However, in some genes, two or four exons fused to a single exon. All the analyzed species, except *C. papaya*, have at least one *CDC20* copy with the 5 exon structure (Figure S2 and Figure S3). The two *C. papaya* genes have the last two exons fused. Remarkably, intron-less *CDC20* genes with all exons fused to a single exon were only found in the *Arabidopsis* clade. Besides the *A. thaliana* genes *AtCDC20.3*, *AtCDC20.4* and *AtCDC20.5*, one intron-less gene was present in *A. lyrata* (AL6G28380). *A. lyrata* has in addition two genes with the only presence of the first intron and one gene with the conserved 5 exon structure. Our analyzes collectively show high conservation of the 5 exon gene structure in the plant *CDC20* gene family although loss of introns, except the first one, can occur in certain gene copies while intron-less genes seem to be unique for Arabidopsis.

In the AtCDC20 isoforms the sequence variations affected also the functional motifs (Figure 1C). Nevertheless the C-box, required for the binding of CDC20 to the APC/C core was conserved in all AtCDC20s. The C-terminal APC/C-binding IR motif was found in AtCDC20.1, AtCDC20.2 and AtCDC20.5, while AtCDC20.3 and AtCDC20.4 terminate with LR residues. Albeit IR is conserved in the animal CDC20/CDH1 proteins, the homologous I/L replacement can be functional as an LR motif is present, for example in the CDC20 of *Pichia stipitis*. Therefore it was likely that all the five *A. thaliana* CDC20 isoforms can potentially interact with the APC/C.

Both the D-box and KEN box degron motifs that can mediate CDC20 destruction by APC/C^CDH1/CCS52^ are present in AtCDC20.1 and AtCDC20.2. AtCDC20.5 contains only the KEN-box and thus still could be a substrate for APC/C^CDH1/CCS52^. In contrast, there are no degrons in AtCDC20.3 and AtCDC20.4, suggesting that the stability of these latter putative proteins is not controlled by the APC/C^CDH1/CCS52^.

The MAD2 and the cyclin binding motifs, as well as a putative BUB1 phosphorylation site in front of the KEN box were conserved in all AtCDC20s (Figure 1C). Additional motifs found in the human CDC20, such as a 2^nd^ MAD2 binding site [25], the CRY-box or metal binding domain were absent in the AtCDC20s. Based on the presence of these motifs, thus all the five AtCDC20s have the potential to interact with the APC/C, MAD2 or mitotic cyclins while only AtCDC20.1 and AtCDC20.2 and perhaps AtCDC20.5 could be subjected to APC/C^CCS52^ mediated degradation control.

### Subcellular localization of AtCDC20s

**Figure 2:**
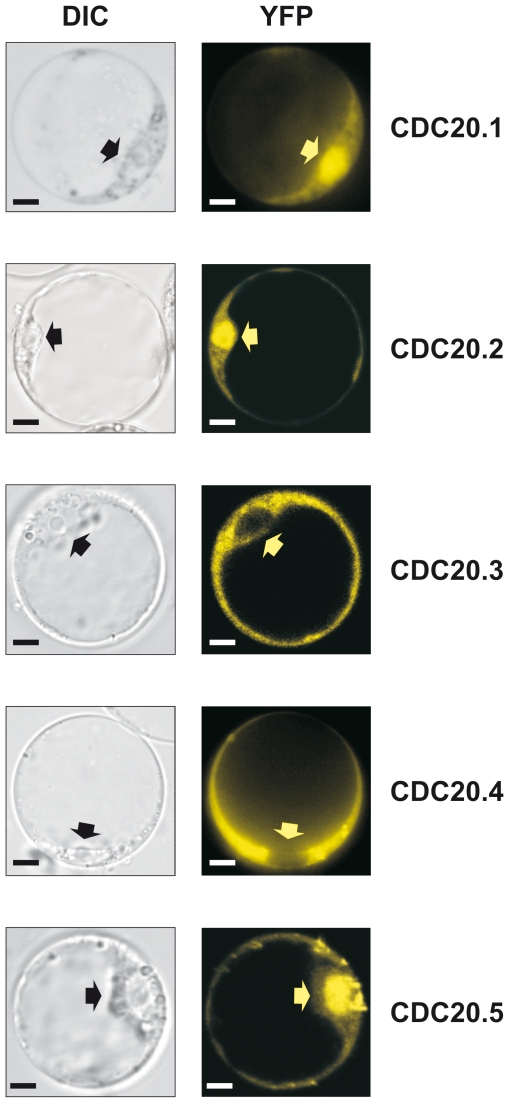
Subcellular localization of the 35S-AtCDC20-YFP fusions in *A. thaliana* protoplasts. DIC: differential interference contrast image; YFP: fluorescence image for detection of the yellow fluorescent protein. Arrows mark the nucleus. Scale bars = 5 µm.

In interphase animal cells, nuclear but also substantial cytosolic localization has been reported for CDC20s [26]. To study whether the Arabidopsis isoforms dispose a uniform subcellular localization, the cDNAs of AtCDC20 isoforms tagged with the yellow fluorescent protein (YFP) were expressed in Arabidopsis protoplasts under the control of the continuously active 35S promoter (Figure 2). AtCDC20.1, AtCDC20.2 and AtCDC20.5 were mainly nuclear, similarly to the animal CDC20 proteins, while AtCDC20.3 and AtCDC20.4 were excluded from the nucleus. The non-uniform localization of the highly homologous isoforms raised thus the possibility that there might be functional differences amongst the isoforms.

### Interaction of AtCDC20.1 and AtCDC20.2 with the APC/C

**Figure 3:**
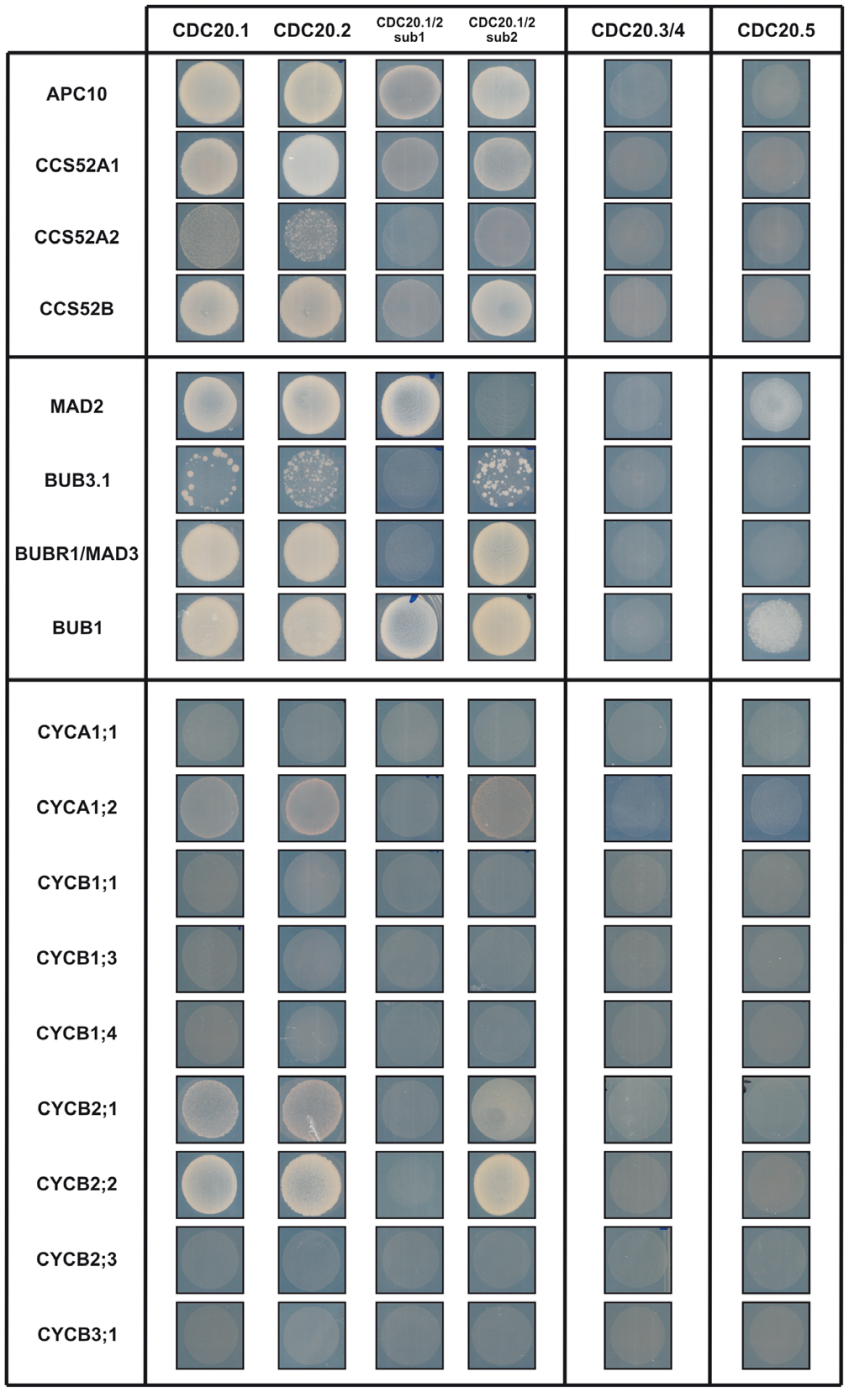
Pair-wise yeast two-hybrid interactions of AtCDC20s with APC subunits, MCC components and mitotic cyclins. Yeast growth is shown at day 6 after transformation. As AtCDC20.1 and AtCDC20.2 and their subdomains interacted similarly with the tested proteins, subdomain interactions are shown as AtCDC20.1/2 indicating either of the two proteins. Similarly AtCDC20.3/4 corresponds to AtCDC20.3 and AtCDC20.4.

Mammalian and yeast CDC20 proteins interact with the APC/C complex as well as with D-box APC/C substrates. Both the CDC20 and CDH1/CCS52 proteins have the C-box and the IR domain for interaction with the APC/C. The latter terminal residues mediate binding to the tetratricopeptide repeat (TPR) subunits (CDC27/APC3, CDC16/APC6 and CDC23/APC8) of the APC/C while the C-box interacts with other APC/C subunits. Previously we have shown in pair-wise yeast two-hybrid (Y2H) assays that HOBBIT, one of the two isoforms of the core APC subunit CDC27, interacted with AtCDC20.1, AtCDC20.2 and AtCDC20.5 as well as with the AtCCS52s, but not with AtCDC20.3 and very weakly with AtCDC20.4 [27]. Here we investigated whether Y2H interaction occurs between AtCDC20s and the docking component, APC10/Doc1 which contributes to substrate recognition and is required for elongation of the ubiquitin chain on the substrate protein [28]. Even though all AtCDC20 isoforms were expressed in yeast as confirmed by Western blot analysis (Figure S4) we detected only the binding of AtCDC20.1 and AtCDC20.2 to APC10 (Figure 3). Studying separately the binding properties of the N-terminal helical part containing the characteristic CDC20 domains (sub1) and the β-propeller WD40 repeat region with the terminal IR sequence (sub2) of AtCDC20.1 and AtCDC20.2 (Figure 1C), we showed that both regions were required for the APC10 interaction, but with a more prominent role of sub2 in the binding (Figure 3).

As CDC20 itself is an APC/C^CDH1^ substrate we studied also how the AtCDC20 isoforms interact with the AtCCS52 proteins. The AtCDC20.3, AtCDC20.4 and AtCDC20.5 showed no interaction with the AtCCS52s. In contrast, AtCDC20.1 or AtCDC20.2 interacted with all the three AtCCS52s with preference for AtCCS52A1 and AtCCS52B. In spite of the presence of the D-box and KEN-box sequences on the N-terminal part, the binding was more significant with the WD40 repeat/IR region of AtCDC20.1 and AtCDC20.2 indicating that the D-box and KEN-box degrons are not the only motifs for activator-substrate interactions.

### AtCDC20.1 and AtCDC20.2 are components of the mitotic checkpoint complex in Arabidopsis

CDC20, MAD2, BUB3 and BUBR1/MAD3 are components of the MCC in animals and yeasts. Recent work by Caillaud et al. [29] demonstrated that the Arabidopsis BUBR1/MAD3 (At2g33560), MAD2 (At3g25980) and BUB3.1 (At3g19590) proteins interacted physically with each other supporting conserved roles of these proteins also in plants. Here we investigated in Y2H pair-wise assays how these proteins interact with the AtCDC20s (Figure 3). Binding of AtCDC20.1 and AtCDC20.2 was detected to MAD2 and BUBR1/MAD3 and a weaker one to BUB3.1. AtCDC20.5 interacted also with MAD2, but not with BUBR1/MAD3 and BUB3.1. AtCDC20.3 and AtCDC20.4 did not bind to any of these proteins.

The binding sites in AtCDC20.1 and AtCDC20.2 for the MCC proteins were delimited with the use of the N-terminal (sub1) and the C-terminal (sub2) regions of AtCDC20.1 and AtCDC20.2. As expected, the MAD2 binding required only the sub1 region where the MAD2 binding site is located (Figure 1C). However, the inability of AtCDC20.3 and AtCDC20.4 to interact with MAD2, despite the conservation of the MAD2 binding site, suggests that, in addition to the consensus MAD2 binding motif, the neighboring regions may also contribute to the binding properties. In yeasts and animals, the MAD2 proteins form a dimer [30]. Likewise, the Arabidopsis MAD2 is able for self-interaction in the Y2H system (data not shown). BUB3.1 and BUBR1/MAD3 interacted only with the sub2 region supporting the involvement of WD40 repeats in the binding.

CDC20 is negatively regulated by BUB1 phosphorylation [31]. A putative BUB1 phosphorylation site was predicted in all AtCDC20s at the N-terminus (Figure 1C). AtCDC20.1 and AtCDC20.2 interacted strongly and AtCDC20.5 weakly with BUB1 (Figure 3). Both sub1 and sub2 regions of AtCDC20.1 and AtCDC20.2 were able to bind BUB1 indicating multiple interaction sites with BUB1 and perhaps the presence of further BUB1 phosphorylation sites in AtCDC20.1 and AtCDC20.2. All these interactions supported the conserved CDC20 cell cycle function for AtCDC20.1 and AtCDC20.2 in the SAC mechanism and the formation of the MCC, while the involvement of the other three isoforms in the mitotic cell cycle events was unlikely.

### Interaction of AtCDC20.1 and AtCDC20.2 with mitotic cyclins

The essential role of APC/C^CDC20^ in yeasts and animals is the degradation of securin and A- and B-type mitotic cyclins during mitosis [6]. Interestingly, there is no obvious securin homolog in the Arabidopsis genome, whereas there are 10 A-type cyclin (*CYCA*) and 11 B-type cyclin (*CYCB*) genes. The expression pattern of most mitotic cyclins is G2-M specific; however, certain mitotic cyclins are also expressed in other phases of the cell cycle [32], [33]. All mitotic cyclins contain the D-box sequence and all the AtCDC20 and AtCCS52 isoforms have the cyclin binding RVL motif. This raised the possibility that the APC/C activators may interact with any of these cyclins and selective degradation of individual cyclins by different APC/C^CDC20^ or APC/C^CCS52^ forms could simply rely on the expression pattern of the genes and the co-existence of activator proteins and mitotic cyclins in a given cell.

In order to test if the CDC20-mitotic cyclin interaction is general or selective we cloned nine Arabidopsis mitotic cyclins (*CYCA1;1*, *CYCA1;2*, *CYCB1;1*, *CYCB1;3*, *CYCB1;4*, *CYCB2;1*, *CYCB2;2*, *CYCB2;3*, *CYCB3;1*) and studied their pair-wise interactions with each AtCDC20 in Y2H assays. In spite of the production of all these plant mitotic cyclins and CDC20s in yeast (Figure S4) only AtCDC20.1 and AtCDC20.2 gave interactions with mitotic cyclins that was, however, restricted to CYCA1;2, CYCB2;1 and CYCB2;2 with high preference for CYCB2;2 and exhibiting weak binding to CYCA1;2 (Figure 3). Such a strong selection of AtCDC20s toward the mitotic cyclins was unexpected and indicated that variations in the highly degenerate D-box sequence (Rxx-LxxxxN/Q) of mitotic cyclins may influence the binding efficiency, or in addition to the RVL motif further sequences contribute to the binding of mitotic cyclins to the AtCDC20s. Using the sub1 and the sub2 regions we showed that the interaction of AtCDC20s with mitotic cyclins requires only the C-terminal sub2 region of AtCDC20.1 or AtCDC20.2. Sub2s of AtCDC20.1 or AtCDC20.2 differ from AtCDC20.3, AtCDC20.4 and AtCDC20.5 at 51 positions, from which 36 are non-homologous amino acid replacements. These variations in the isoforms may influence the cyclin binding properties of AtCDC20s and likely the RVL motif alone is not sufficient for binding of mitotic cyclins.

These results suggests that the high number of A- and B-type cyclins and variations in the D-box sequences might have evolved in parallel with the AtCDC20 and AtCCS52 isoforms to ensure degradation of specific mitotic cyclins at given stages of the cell cycle or plant development.

### Expression of *AtCDC20*s during the cell cycle and plant development

**Figure 4:**
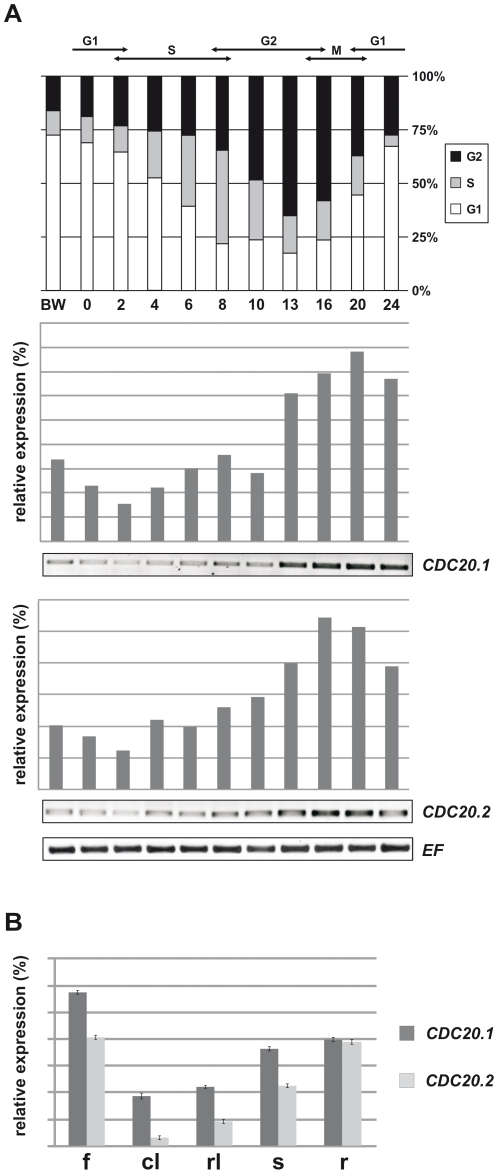
Expression of *AtCDC20.1* and *AtCDC20.2* in synchronized Arabidopsis cell culture and different plant organs. (**A**) Expression of *AtCDC20.1* and *AtCDC20.2* during the cell cycle. The diagram shows the progression of the cell cycle after aphidicolin block and distribution of the cells at distinct phases of cell cycle (G1, S, G2, M) (BW, before aphidicolin wash; 0–24, hours after removal of the aphidicolin). The RT-PCRs show the relative expression of *AtCDC20.1* and *AtCDC20.2* genes normalized to the expression of *elongation factor* (*EF*), used as a constitutive marker, in function of time (0–24 hours) after the release from the aphidicolin block. (**B**) Relative expression of the *AtCDC20.1* and *AtCDC20.2* genes in flowers (f), cauline leaves (cl), rosette leaves (rl), stems (s) and roots (r) by RT-qPCR normalized to the expression level of the *EF* constitutive marker.

We studied the expression pattern of the five *AtCDC20* genes in aphidicolin synchronized Arabidopsis cell cultures with RT-PCR (Figure 4A). Similarly to the yeast and animal *CDC20s*, *AtCDC20.1* and *AtCDC20.2* expression started to rise in the S-phase and peaked in the M-phase with a non-negligible basic expression level also in the other cell cycle phases. The expression of the two genes was overlapping and suggested largely redundant functions of these two isoforms. Unexpectedly, no or background expression levels were detected for the other three *AtCDC20s* (data not shown) indicating that these genes may have no or at most only minor cell cycle functions.

The CDC20 functions are linked to mitotic and meiotic cells. Expression of the *AtCDC20* genes was expected in the meristems, organ primordia and young developing organs where plant cells divide. The existing Arabidopsis microarray data from the Genevestigator [34] or the Arabidopsis eFP browser [35] do not distinguish between the AtCDC20.1 and AtCDC20.2 isoforms. Nevertheless, the absolute signal threshold of overall expression for the *AtCDC20.1* and *AtCDC20.2* was 852.71 (Arabidopsis eFP browser) versus the background signal levels of *AtCDC20.3* (6.26), *AtCDC20.4* (1.68) and *AtCDC20.5* (7.26) in the tested conditions indicating that *AtCDC20.1* and *AtCDC20.2* are the key *CDC20* genes in Arabidopsis.

By using specific oligos for each isoforms, we intended to confirm the microarray data with RT-qPCR and to study whether the *AtCDC20.1* and *AtCDC20.2* genes are expressed on a similar or specific manner. RNA was extracted from flowers, cauline and rosette leaves, stems and roots for cDNA synthesis. In agreement with the background expression levels of *AtCDC20.3*, *AtCDC20.4* and *AtCDC20.5* on the microarrays, we did not detect the expression of any of them. In contrast, we confirmed the activity of both *AtCDC20.1* and *AtCDC20.2* in these organs (Figure 4B).

**Figure 5:**
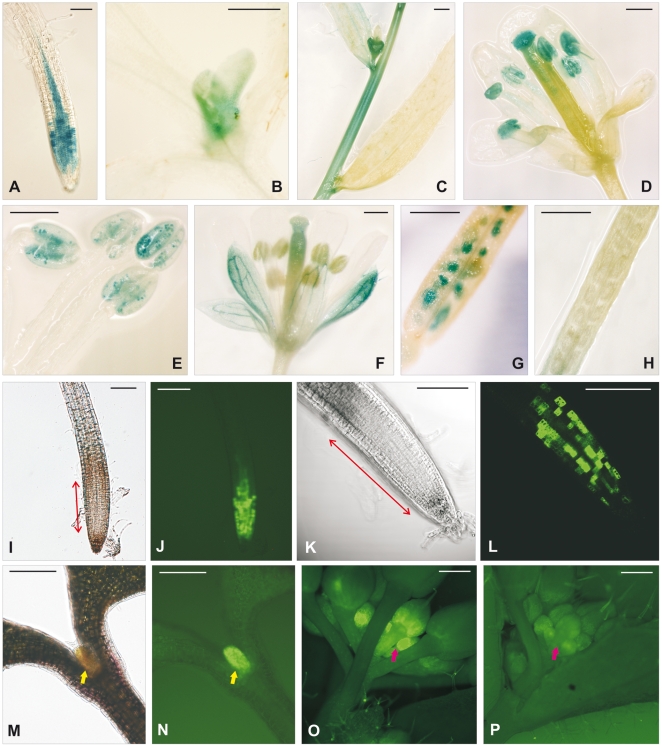
Temporal and spatial expression of the *AtCDC20.1* and *AtCDC20.2* genes during plant development. (**A**) to (**H**) Expression patterns of the *AtCDC20.1-GUS* and *AtCDC20.2-GUS* lines. Expressions of *AtCDC20.1* in the primary root (**A**), leaf primordium (**B**) and in young stem segment (**C**) was identical to that of *AtCDC20.2*. Expression of *AtCDC20.1* in the flower bud (**C**), anthers (**D**), pollen grains (**E**) and developing seeds (**G**) was specific while *AtCDC20.2* was expressed in the sepals and style (**F**), but not in the silique (**H**). Blue color marks the β-glucuronidase activity of the GUS reporter gene. (**I**) to (**P**) Expression patterns of the *AtCDC20.1-GFP* or *AtCDC20.2-GFP* lines. DIC image of the root meristem at lower (**I**) and higher (**K**) magnifications and that of the leaf primordium (**M**). *AtCDC20.1-GFP* expression in the root meristem at lower (**J**), and higher (**L**) magnifications, in the leaf primordium (**N**) and in the flower bud (**O**). The expression pattern of *AtCDC20.2-GFP* was overlapping with that of *AtCDC20.1-GFP* with the exception of the flower buds where the *AtCDC20.2-GFP* signal was at the background level (**P**). Bright green color reflects the GFP fluorescence, the size of the root meristem is indicated with two-way red arrows, yellow arrow marks the leaf primordium and the red one the flower buds. Scale bars = 200 µm.

To study *in situ* the expression of these isoforms in different organs during different stages of plant development, we fused the GUS reporter gene with the start codon of *AtCDC20* genes preceded with the promoter region. Surprisingly none of these constructs resulted in GUS activity. Therefore, in addition to the putative promoter region, the first exon and intron together with the first codon of the 2^nd^ exon of *AtCDC20.1* and *AtCDC20.2* were fused to *GUS*. These translational fusion constructs resulted in GUS activity that was monitored in 10–12 independent transgenic *A. thaliana* lines per construct in the T1 and T2 populations. The *AtCDC20.1-GUS* and *AtCDC20.2-GUS* lines displayed identical expression pattern in the vegetative organs presented for the example of *AtCDC20.1-GUS* in Figure 5. The GUS activity was visible in the root meristem (Figure 5A) where the spotty expression pattern was in line with cell cycle regulation of *AtCDC20.1* and *AtCDC20.2* genes. They were also redundantly expressed in leaf primordia (Figure 5B) and in young stem segments (Figure 5C). On the contrary, the expression of *AtCDC20.1* and *AtCDC20.2* was different and complementary during the flower development. *AtCDC20.1*, but not *AtCDC20.2*, was expressed in the flower buds (Figure 5C), stigma and anthers (Figure 5D). In the anthers the expression was localized to the pollen grains (Figure 5E). The *AtCDC20.2* expression was detected in the sepals, particularly in the vascular tissue and weakly in the style (Figure 5F). Expression of *AtCDC20.1* was also detectable during seed development (Figure 5G) but not that of *AtCDC20.2* (Figure 5H).

The expression pattern of *AtCDC20.1* and *AtCDC20.2* was also verified with promoter-ORF-GFP translational fusions using the genomic DNA comprising the same promoter regions as in the GUS constructs and the entire coding region, including the introns, fused to GFP. Likewise the GUS staining, localizations of the *AtCDC20.1* and *AtCDC20.2* driven GFP signals were largely overlapping. Both genes as shown for *AtCDC20.1* were expressed in the root meristem (Figure 5I,J) where the spotty cell cycle regulated gene expression was even better visible (Figure 5K,L) and was without the background GUS signal in the vascular tissue (Figure 5A,J). Likewise the root, *AtCDC20.1* and *AtCDC20.2* were similarly expressed in the leaf primordia (Figure 5M,N). The AtCDC20.1-GFP fluorescence was present in the young flower buds (Figure 5O) where the detection of AtCDC20.2-GFP was at background level (Figure 5P). Because of the high autofluorescence of the anthers, the GFP fusions could not be used for evaluation of gene expression. Although both the RT-qPCR data and the GUS-staining support *AtCDC20.2* expression in the flowers, AtCDC20.2-GFP protein was not detectable in the sepals indicating that the fusion protein might be degraded. Collectively, the GUS and GFP expression data support redundant functions for AtCDC20.1 and AtCDC20.2 in root and leaf development but divergent ones in the flower and specific role for AtCDC20.1 in seed development.

### Simultaneous down-regulation of *AtCDC20.1* and *AtCDC20.2* has a negative effect on plant growth and results in male sterility

For the functional analysis of *AtCDC20* genes, we investigated the following T-DNA mutant lines: *cdc20.1-1* (SAIL813A03, promoter), *cdc20.1-2* (GK568G01, 4^th^ exon), *cdc20.2-1* (SALK114279C, 3^rd^ intron), *cdc20.2-2* (SALK136724, 4^th^ exon), *cdc20.3* (SALK002496, exon), *cdc20.4* (GK702F07, promoter), and *cdc20.5* (SALK083223, exon) mutants. After generation of homozygous lines for each mutant, their phenotype and development were compared to wild type plants. None of these mutants displayed obvious phenotypic alterations. This was not surprising in the case of the *cdc20.3, cdc20.4* and *cdc20.5* mutants as these genes do not show expression. In contrast, mutations in the *AtCDC20.1* and *AtCDC20.2* genes were expected to perturb the mitotic cycle and to result in severe phenotypic alterations. The lack of phenotype in these mutants, together with the largely overlapping expression pattern of *AtCDC20.1* and *AtCDC20.2*, suggested redundant functions of these isoforms.

**Figure 6:**
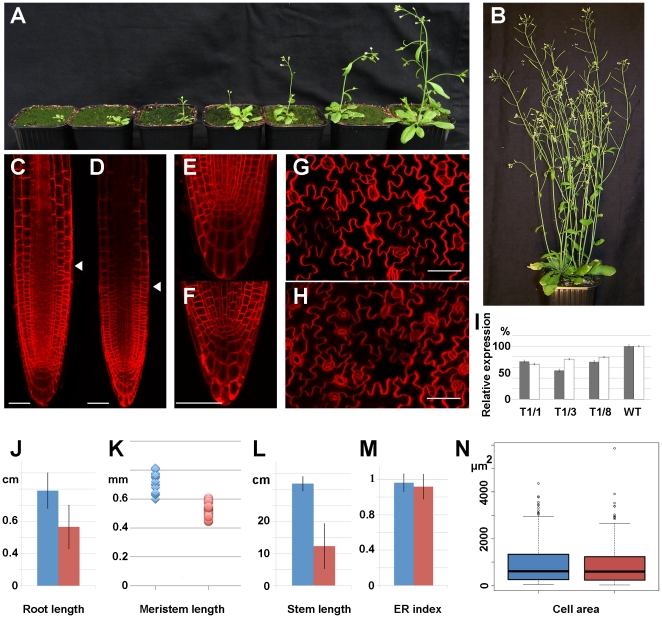
Plant phenotypes by simultaneous down-regulation of *AtCDC20.1* and *AtCDC20.2* with RNA interference. (**A**) Retarded growth of the RNAi plants compared to a wild type plant (**B**) of the same age. (**C–H**) Confocal image of FM4-64 stained control root meristem (**C**) and RNAi root meristem (**D**), control root tip (**E**), RNAi root tip (**F**), control leaf (**G**) and RNAi leaf regions (**H**). Scale bars = 50 µm. (**I**) Relative expression of the *AtCDC20.1* (grey) and the *AtCDC20.2* (white) genes in the flowers of three different T1 RNAi lines (T1/1, T1/3, T1/8) in respect to wild type plants by RT-qPCR normalized to the expression level of *EF*. (**J**) Root length. (**K**) Meristem size. (**L**) Stem length. (**M**) Endoreduplication index. (**N**) Area of pavement cells. Arrowhead in (**C**,**D**) marks the root meristem-elongation zone border. Blue color in (**J–N**) corresponds to the control plants while the red one to the RNAi plants.

As the *AtCDC20.1* and *AtCDC20.2* genes are only separated by 1 kb, generation of double mutants had low feasibility. In addition, the double null mutant was expected to be lethal due to the essential role of CDC20 in the cell cycle. Therefore, we knocked down gradually and simultaneously the expression of both *AtCDC20.1* and *AtCDC20.2* by RNA interference (RNAi) using a region that was conserved in both genes and absent in other genes. 86 transgenic plants were obtained from five biological repeats. These RNAi plants, depending on the degree of the RNAi effect, were smaller and less developed (Figure 6A) than the wild type plants that were germinated at the same time (Figure 6B). Nevertheless, beside severe delays from several weeks up to several months in their development, the RNAi lines displayed no morphological aberrations in their vegetative organs.

Correlation between the phenotypes and gene expression levels was investigated in selected RNAi lines by RT-qPCR (Figure 6I). The *AtCDC20.1* transcript levels were reduced by 30–45% and the *AtCDC20.2* transcript levels by 20–35% supporting the link between delayed plant growth and reduced expression of the *AtCDC20.1* and *AtCDC20.2* genes.

In the RNAi lines the root length was significantly reduced (Figure 6J). As *AtCDC20.1* and *AtCDC20.2* are expressed in the root meristem we studied how down-regulation of these genes affects the size and organization of the root meristem. The root meristem was significantly smaller in RNAi plants than in the control ones (Figure 6C,D and K) while the root patterning was not affected (Figure 6E,F). Thus lower activity of the meristem producing fewer cells could be the cause of reduced root growth.

The *AtCDC20.1* and *AtCDC20.2* genes are also active in the shoot apical meristem and therefore their down-regulation is expected to diminish the size of this meristem as well. Accordingly, in the RNAi lines, the size of the aerial part was strongly reduced (Figure 6L) and the leaves were smaller (Figure 6A). The Arabidopsis leaf growth depends on the meristematic activity, the cell number as well as on the formation of large polyploid cells arising from endoreduplication cycles. Measuring the ploidy levels of the control and RNAi leaves by flow cytometry revealed no differences in the endoreduplication index which is calculated on the basis of distribution of cell populations with different ploidy levels (Figure 6M). The area of leaf pavement cells in the control and RNAi lines was similar (Figure 6G,H) and the Kruskal-Wallis rank sum test confirmed that there was no significant difference (P = 0.4545) between them (Figure 6N) indicating that reduced leaf size is the consequence of lower cell number in the RNAi lines.

**Figure 7:**
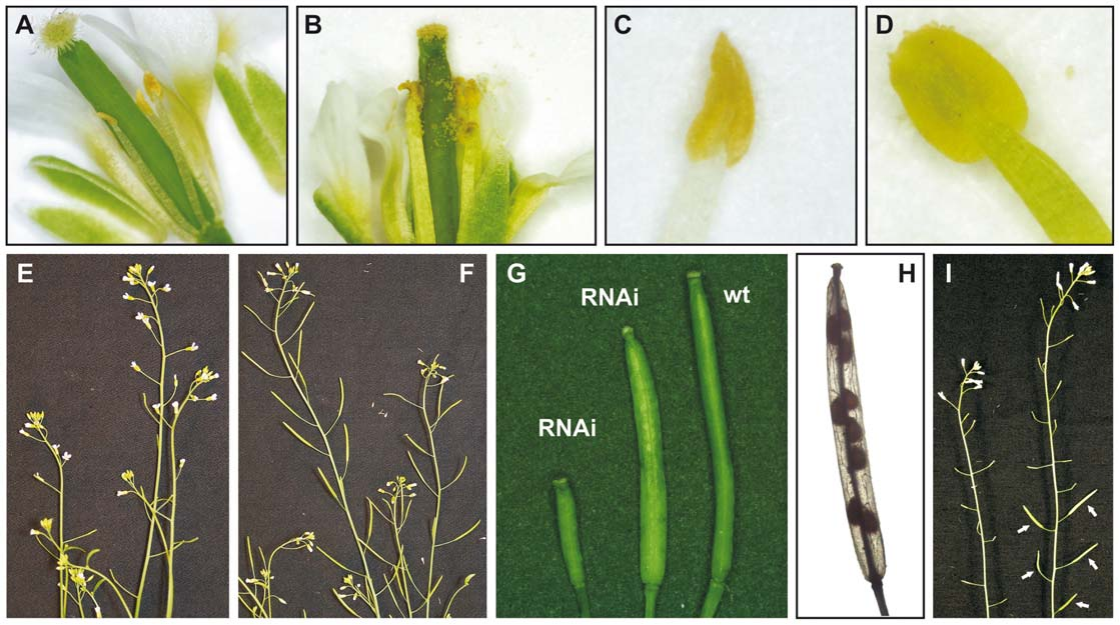
Phenotypes of flower organs induced by down-regulation of *AtCDC20.1* and *AtCDC20*.2. (**A**) Flower of an RNAi plant and (**B**) wild type flower. (**C**) Collapsed empty anther in an RNAi plant and (**D**) wild type anther. (**E**) Infertile RNAi plants with aborted silique development versus (**F**) wild type flowers and siliques. (**G**) Siliques of wild type and the RNAi lines. (**H**) Seed abortion in an RNAi silique. (**I**) Cross pollination of the RNAi flowers with wild type pollen restores silique development (arrows).

During the reproductive stage, the transgenic lines had consistently a gradient of anomaly in fertility. While the flower structure appeared to be normal, in many RNAi lines the stigma was free of pollen (Figure 7A) in contrast to pollen-covered stigma in wild type flowers (Figure 7B). Unlike the wild type anthers (Figure 7D), the anthers in the RNAi lines were collapsed (Figure 7C). In these collapsed anthers the pollen production was reduced or completely abolished. In the absence of pollen grains, the silique development was blocked (Figure 7E) in remarkable contrast to wild type silique development (Figure 7F). The RNAi lines with reduced fertility developed shorter siliques than the wild type plants (Figure 7G) and were only partially filled with seeds (Figure 7H). The abortion of embryo development was indicative of male sterility. Indeed, cross-pollination of the pollen-free RNAi flowers with wild type pollen restored the fertility resulting in normal silique and seed development (Figure 7I).

## Discussion

### Two of the five Arabidopsis *CDC20* genes encode authentic and functional CDC20 proteins

The five gene copies in Arabidopsis suggested to us that the different isoforms could have novel or complementary roles during plant development. All AtCDC20 isoforms contain the known structural motifs to interact with the APC/C and mitotic cyclins. In the absence of securin in plants, the major targets of APC/C^CDC20^ are the mitotic cyclins. Thus, it seemed plausible that each isoform controls the degradation of a specific subset of cyclins. The specificity could be provided either by differential expression of the *AtCDC20*s or by the selective interaction with cyclins. However, in contrast to our initial assumptions, our work demonstrates that only two isoforms, AtCDC20.1 and AtCDC20.2 play roles in the cell cycle.

Protein interactions with APC subunits, components of MCC and mitotic cyclin substrates were only demonstrated consistently for AtCDC20.1 and AtCDC20.2. Furthermore, our gene expression studies and the publicly available transcriptome data (Genevestigator, Arabidopsis eFP browser) failed to detect expression of the *AtCDC20.3*, *AtCDC20.4* and *AtCDC20.5* genes above background levels.

Phylogenetic analysis of the plant CDC20 proteins failed to identify distinct CDC20 subclasses in contrast to evolution of the CCS52A and CCS52B subclasses of CCS52 proteins in plants [18], [36]. In the *CDC20* gene structures, the presence of four introns and their respective positions were well conserved. Nevertheless, several genes have lost one or more of these introns but strikingly the first intron has always been maintained except in *AtCDC20.3*, *AtCDC20.4*, *AtCDC20.5* and one of the *A. lyrata* genes which contained no introns. The promoter-GUS analysis of the *AtCDC20.1* and *AtCDC20.2* genes revealed that the putative promoter region alone was insufficient for GUS expression. Nonetheless, significant GUS coloration was obtained when the first intron was also present in the fusion construct. Within this intron, an 80 bp long sequence has been conserved (data not shown) that might be crucial for the expression and common regulation of the *AtCDC20.1* and *AtCDC20.2* genes.

The intron-less *CDC20* genes are unique to the *Arabidopsis* clade. The formation of intron-less *AtCDC20.3*, *AtCDC20.4* and *AtCDC20.5* genes could have occurred via insertion of reverse transcribed mRNAs into the genome [37]. Such retroposed genes typically do not contain the promoter and introns of the parental gene but sometimes have a recognizable poly-adenine tail (if it has not been decayed). Retroposed genes can constitute novel genes by the recruitment of regulatory elements and acquiring novel functions via gene fusion resulting in expressed and functional “retrogenes”. Nevertheless, frequently they are “pseudogenes” often having diagnostic frame disruptions, stop codons or interspersed repeats [37]. The *AtCDC20.3*, *AtCDC20.4* and *AtCDC20.5* genes are present on the same chromosome, thus they result probably from a single retrotranscription event followed by multiplication of the retroposed gene. The *AtCDC20.3*, *AtCDC20.4* and *AtCDC20.5* genes have no stop codons or repeats indicating that the retrotranscription event was relatively recent. In the absence of promoter/enhancer activity at the site of insertion and lacking the promoter region and the first intron of the parental gene, the *AtCDC20.3*, *AtCDC20.4* and *AtCDC20.5* genes appear to be inactive. Nevertheless, we cannot exclude the possibility that under specific conditions these retrogenes might have cryptic expression and yet undiscovered functions.

### AtCDC20.1 and AtCDC20.2 are indispensable for normal plant development and fertility

*AtCDC20.1* and *AtCDC20.2* are expressed in all plant organs containing dividing cells. Their cell cycle regulated expression was clearly visible in the root meristem. In contrast to the overlapping expression pattern in the vegetative organs, expression of the two genes was clearly different in the flower where *AtCDC20.1* was expressed in the flower buds and the pollen grains while *AtCDC20.2* in the sepal vasculature. Expression in the pollen suggests a role for AtCDC20.1 also in the meiotic cell cycle. The single T-DNA insertion mutants of *AtCDC20.1* or *AtCDC20.2* showed no visible phenotypes. Therefore functional analysis of these genes was carried out by simultaneous diminution of the *AtCDC20.1* and *AtCDC20.2* mRNA levels by RNAi. In accordance with the vital function of CDC20 in the cell cycle, the RNAi lines exhibited only moderate levels of reduction in the *AtCDC20.1* and *AtCDC20.2* transcript levels. This reduction was comparable to downregulation of *CCS52A* in *M. truncatula* where RNAi lines were recovered at most with 40% reduction [19]. This relatively mild down-regulation of the *AtCDC20.1* and *AtCDC20.2* genes caused nevertheless a severe delay in the plant development indicating that *AtCDC20.1* and *AtCDC20.2* transcripts might be limiting for cell cycle progression which slow down cell proliferation. Moreover, the reduced or completely abolished fertility of the RNAi lines supports also roles for AtCDC20.1 and AtCDC20.2 in meiosis. Though only the *AtCDC20.1* gene expression was detected in the pollen grains, the absence of male sterility in the *cdc20.1-2* insertion mutant indicates that AtCDC20.2 may complement the mutant gene function.

### Interaction of AtCDC20.1 and AtCDC20.2 with the APC/C and mitotic cyclins

The minimal ubiquitin ligase module of the APC/C comprises APC2 and APC11. These two subunits, together with the E2 enzyme, are sufficient for ubiquitination reactions but lack substrate specificity [5]. APC10 is required for the ubiquitination process of substrate proteins as well for the substrate recognition [28]. TPR domains in CDC23/CDC27/CDC16 recruit CDH1 and CDC20 to the APC/C. Previously we have shown the interaction of AtCDC20.1, AtCDC20.2 and AtCDC20.5 with the TPR subunit CDC27b (HOBBIT) in Arabidopsis [27]. This study revealed the direct binding of AtCDC20.1 and AtCDC20.2 to APC10. This suggests that the IR motif in the CDC20 and the CCS52 proteins anchors the activators on CDC27 and their binding to the APC/C is further strengthened by their interaction with APC10. These bindings likely provoke conformational changes in the APC/C^CDC20^ complex which may facilitate the presentation of the CDC20-bound substrates for ubiquitination by the APC/C catalytic centre.

The A-type cyclins are degraded by APC/C^CDC20^ in early M-phase at the breakdown of the nuclear envelop [38], while the B-type cyclins are degraded at the onset of anaphase. AtCDC20.1 and AtCDC20.2 are 99% identical and their interaction with the tested mitotic cyclins was the same. The mitotic cyclin interactions were, however, surprisingly restricted, as binding occurred only with three out of the nine tested cyclins. The binding of APC/C activators and mitotic cyclins necessitates the presence of the RLV cyclin binding motif in the activator and the RxxLxxxxN/Q D-box sequence in the mitotic cyclin. The RLV motif is conserved in all AtCDC20 and AtCCS52 isoforms and the D-box is present in all mitotic cyclins. One can consider that the selective interaction of AtCDC20s with the mitotic cyclins is the consequence of the D-box sequence divergences. On the other hand, the inability of AtCDC20.3, AtCDC20.4 and AtCDC20.5 to interact with mitotic cyclins indicates that the RLV motif alone might not be sufficient for cyclin binding. Most likely the non-homologous amino acid replacements along the WD40 repeats influence the cyclin binding properties of AtCDC20s.

The constitutive overexpression of a non-degradable mitotic cyclin was shown to provoke strong perturbation of the mitotic cycle [39]. If AtCDC20s target only a fraction of mitotic cyclins, then how the others are degraded and how normal cell cycle progression is achieved? In Arabidopsis, at least 20 mitotic cyclins have been predicted, however it is still elusive how many of them and which ones are active in the meristems and dividing cells. The actual number of functional cyclins could be less than the predicted number of genes. Cell cycle regulated expression of the plant specific CDH1-type APC/C activator *AtCCS52B* largely overlaps with that of *AtCDC20.1* and *AtCDC20.2* [32]. AtCCS52B interacts also with mitotic cyclins and therefore AtCCS52B could contribute to mitotic cyclin degradation during M-phase, while mitotic exit and mitotic cyclin destruction during G1 phase could be mediated by AtCCS52A1 and AtCCS52A2 [32]. However, none of the investigated cyclins showed Y2H interactions with any of the AtCCS52s (data not shown), leaving the question open for their proteolytic regulation. Recently Cdc20 and the D-box independent recruitment of mitotic cyclins to APC/C has also been reported in human cell cultures [40]. This raises the possibility that similar mitotic cyclin-APC/C interactions occur in plants which may lead to mitotic cyclin ubiquitination and degradation. Moreover, it cannot be excluded that the interactions of the Arabidopsis CDC20s with the mitotic cyclins might be different in the plant and yeast cells as altered “sampling sensitivity” in Y2H has already been described [41].

### Formation of MCC and chromosome separation

The fidelity of chromosome segregation during mitosis is controlled by the spindle assembly checkpoint. Until the proper bipolar attachment of chromosomes to the spindle microtubules, the SAC mechanism arrests the cell cycle progression by the inhibition of APC/C via the sequestration of CDC20 in the MCC. In Arabidopsis, the BUBR1/MAD3, BUB3.1 and MAD2 homologues have a conserved role in SAC [29]. Here we show that AtCDC20.1 and AtCDC20.2 interact with BUBR1/MAD3, BUB3.1 and MAD2 to form the MCC. AtCDC20.5 also interacts with MAD2 but not with BUBR1/MAD3 and BUB3.1. However, in light of the absence of *AtCDC20.5* expression in dividing cell suspension cultures or in dividing cells of plant organs, it is rather unlikely that AtCDC20.5 takes part in MCC *in vivo*.

Interestingly, the conserved MAD2 binding domain alone was not sufficient for MAD2 binding since AtCDC20.3 and AtCDC20.4 displayed no interaction with MAD2 in spite of the presence of a MAD2 binding site. As the WD40 region (sub2) has no role in MAD2 binding, likely other N-terminal regions that are common in AtCDC20.1, AtCDC20.2 and AtCDC20.5 (e.g. the CPL, KEN, QLAE motifs) may contribute to the MAD2 interaction. BUBR1/MAD3 binds to the sub2 domain of AtCDC20.1 and AtCDC20.2. In this region AtCDC20.3 and AtCDC20.4 differ from the BUBR1-binding isoforms in five non-homologous amino acid replacements (K/N, Q/K, S/V, N/T, L/P) that may abolish the interaction.

During the SAC process, the separase activity is blocked primarily by securin, an inhibitor of the separase enzyme in yeast and animals. However, phosphorylation of separase by mitotic CDKs can also block the enzyme activity [42]. After the bipolar spindle attachments on the kinetochores, the separase becomes active and cleaves the cohesion complex allowing segregation of the chromosomes [7]. The dynamic role of separase (AESP - At4g22970) on the cleavage of cohesin (At3g54670) during meiosis has also been demonstrated in Arabidopsis [43]. On the other hand the lack of securin indicates that in plants the phosphorylation of the separase by CYCLIN B dependent CDK1 (CDKA1 - At3g48750) might be the separase inhibiting mechanism. Following the correct spindle attachments, AtCDC20.1 and AtCDC20.2 are released from the inhibitory MCC and APC/C^AtCDC20.1/2^ provokes destruction of CYCLIN B thereby inactivating CDK1. Thus, the separase becomes active and cleaves the cohesion complex leading to chromatide separations and cell divisions. At the anaphase, the APC/C^AtCCS52^ complexes degrade the AtCDC20s and provoke the mitosis exit. Based on the expression patterns of *AtCCS52s* [32], AtCDC20s might be principally degraded by the plant specific APC/C^AtCCS52B^.

*AtCDC20.1* has specific expression in flower buds and in the anthers, where the meiotic division of pollen mother cells occurs. The involvement of AtCDC20.1 in meiosis is further supported by the functional analysis of the Arabidopsis separase. The RNAi lines of AESP disposed the same anther defect with male sterility and aborted siliques [43] as the *AtCDC20.1/2* RNAi lines. Thus, the separase activity of Arabidopsis during meiosis can be driven by the AtCDC20.1 via the CYCLIN B dependent CDK1 phosphorylation. In contrast to the male sterility, the meiotic formation of oocytes in the carpel was undisturbed in the *AtCDC20.1/2* RNAi plants and their crosspollination with wild type pollen resulted in normal seed development. A role of the APC/C^CDC20^ in male gametophyte development is in agreement with the previously demonstrated differential importance of the APC2, APC6/CDC16, APC8 and APC13 subunits in male or female gametophyte formation [22], [44], [45]. Similarly, APC/C is not required for the normal chromosome separation at the anaphase I of meiosis I during the development of *Xenopus* oocytes [45] but CDC20 is critical for correct formation of female gametes [46]. All these findings point to distinct meiotic regulations of the meiotic anaphase I in the male and female gametophytes in plants and animals. However, it remains elusive why the different APC/C subunits affect differently the formation of male and female gametophytes.

In conclusion, our work demonstrates that only two of the five CDC20 isoforms, AtCDC20.1 and AtCDC20.2 perform conserved and redundant functions in the mitotic and meiotic cell cycle. These isoforms mediate selective degradation of certain A- and B-type mitotic cyclins. By interacting with MAD2, BUBR1/MAD3 and BUB3.1 they can be component of the MCC which restrains their activity during SAC. The major mechanism controlling securin activity is likely provided by AtCDC20.1 and AtCDC20.2 which after their liberation from MCC, inhibit CDK1 by CYCLIN B degradation. This work, besides demonstrating conserved CDC20 functions in the M-phase, raises further questions, primarily how the rest of mitotic cyclins are degraded in Arabidopsis and whether other plants operate with similarly numerous CDC20 and cyclin isoforms. Moreover, further studies are required for elucidation of the cooperative actions of CDC20s and CCS52s in APC/C activities during the cell cycle and plant development.

## Materials and Methods

### Transfection of Arabidopsis protoplasts

For transient expression of proteins, AtCDC20 coding sequences were cloned under the control of 35S promoter in the pRT104 vector containing YFP [47]. A cell suspension culture derived from *A. thaliana* ecotype Columbia seedlings was grown in Murashige and Skoog (MS) liquid media containing sucrose (30 g/L), kinetin (14 µg/L) and 2,4-dichloro-phenoxy-acetic acid (2,4-D) at 23°C with 135 rpm under continuous light. The culture was weekly subcultured (15 mL culture in 85 mL fresh medium). Protoplasts were prepared by treating 40 ml of three-day-old cell cultures with cell wall digesting enzymes (cellulase Serva R10 0.01 g/mL, macerozyme Yakult 0.002 g/mL) in MS (4.13 g/L), containing 0.34 M glucose and 0.34 M mannitol (pH 5.5) for 3 to 5 hours at room temperature in the dark. Cells were centrifuged and washed with the culture medium (MS 4.13 g/L, glucose 0.16 M and mannitol 0.16 M, pH 5.5). Protoplasts were then separated from the debris on a sucrose cushion (MS 4.13 g/L and sucrose 0.28 M, pH 5.5) by centrifugation at 1000 rpm for 5 minutes. For transformation, 15 µg of plasmid DNA was added to 10^6^ protoplasts and incubated for 20 min in the dark (PEG 6000 25% w/v, mannitol 0.45 M, calcium nitrate 0.1 M, pH 9). Then protoplasts were rinsed with 0.275 M Ca(NO_3_)_2_ and incubated in the culturing medium overnight in the dark. One day after transfection, protoplasts were observed by confocal microscopy.

### Synchronization of cell cycle in suspension cultured Arabidopsis cells

An *A. thaliana* ecotype Landsberg erecta cell suspension culture [32] was maintained by weekly subculturing in MS medium pH 5.7 supplemented with 3% w/v sucrose, 0.5 mg/L NAA and 0.05 mg/L kinetin. For reversible G1/S blockage, 8-day-old cultures were centrifuged for 5 min at 1500 rpm. Cells were resuspended in fresh MS medium and cultured for 8 hours. Then, aphidicolin was added to the cells at a final concentration of 5 µg/mL for 18 hours. Then aphidicolin was removed by washing the cells with fresh medium lacking hormones and sucrose in two hour intervals for four times (leaving the cells first for 5 min and then three times for 20 min in the washing solution). Finally, the cell pellet was resuspended in fresh MS medium. Samples were taken before wash (BW), after the washes (0 h), and at 2, 4, 6, 8, 10, 13, 16, 20 and 24 hours of incubation. The cell cycle progression was followed by flow-cytometry analysis of DAPI stained nuclei using an ELITE ESP machine (Beckman-Coulter).

### RT PCR, RT-qPCR

For cDNA synthesis, total RNA was isolated from Arabidopsis flowers, cauline and rosette leaves, stems and roots by the RNeasy plant mini kit (Qiagen). RNA was also isolated from Arabidopsis synchronized cell cultures at different time points after release of the aphidicolin blockage. To remove traces of genomic DNA in RNA samples, equal amounts of total RNA were treated with DNase (FPLC pure; Amersham) that was subsequently heat inactivated. cDNAs were synthesized by reverse transcription using Powerscript Reverse Transcriptase (Clontech), RNase inhibitor (RNasin, Promega) and oligo-dT primers and used in PCR after dilution (EUROBIO TAQ polymerase). *AtCDC20s* were amplified in 25 cycles (94°C for 30 s, 55°C for 30 s, and 72°C for 1 min), the *elongation factor* (*EF-At5g60390*), serving as constitutive marker, was amplified in 20 cycles. PCR products were analyzed by the Fisher Scientific Bioblock gel-documentation system. The cDNAs from the plant organs were analyzed by the SYBR® Green-Based Detection system (Applied Biosystems) for real-time qPCR. The qPCR diagrams in Figure 4B and 6I show the average values of three biological replicates. The following PCR primers were used: 5′ cgggtttacacagaatcagctc, 5′ ctgtatcatgggtttccttgtccgtc (tissue specific expression for *AtCDC20.1*); 5′ cactttcttcccaggaaacc, 5′ gaacttaccgctgcagtc, 5′ tcagctcacactttggaagtat, 5′ atatgcacttttcttgtcactac (mutant and RNAi analyses for *AtCDC20.1*); 5′ tcagcttacactttggaagtac, 5′ gtttctttttgtaacaatcaatggg (tissue specific expression for *AtCDC20.2*); 5′ aatggatgcaggtttgaatcgg, 5′gtgaacttaccgttgcagat, 5′ cttcagcagcaggagacgagac, 5′ aatatatagtttctttttgtaacaatcaa (mutant and RNAi analyses for *AtCDC20.2*); 5′ cattactatggagccaaagg, 5′ catctatacctgatgcgaatg (*AtCDC20.3*); 5′ cattattatggagccaaagt, 5′ catgcagtcaaaagctaaag (*AtCDC20.4*); 5′ tggatgcacctggaattgc, 5′ ctgagagtctcgtcaccg (*AtCDC20.5*); 5′ggtggtattgacaagcgtg, 5′ gatttcatcgtacctagcc (*EF*).

### Yeast two-hybrid pair-wise assays

For the Y2H pair-wise interactions pGADT7 (bait) and pGBKT7 (prey) vectors (Clontech) were used for cloning, which were modified for GATEWAY® recombination cloning technology (Invitrogen). The cDNA clones of the investigated genes were obtained by PCR amplification from *A. thaliana* young seedling and cell culture cDNAs with the use of specific oligos and the high-fidelity Phusion enzyme (FINNZYMES). For truncated “sub1” clones, the coding sequence of the first 120 amino acids was used for AtCDC20.1 and the first 111 amino acids for AtCDC20.2. For “sub2” clones, the coding sequence of amino acids 121–457 for AtCDC20.1 and amino acids 112–447 for AtCDC20.2 was included (Figure 1C). The Y2H interaction studies were done according to the protocol of the manufacturer (Clontech - Yeast Protocols Handbook). Interactions were obtained by co-transformation of the *Saccharomyces cerevisiae* yeast strain AH109 with the bait and the prey constructs and selected on SD-WLHA medium (Clontech) which imposed a strong double selection for interactions with the *HIS3* and *ADE2* markers. Strength of the interactions was estimated on the basis of yeast growth on plates: yeast growth within 3 days was qualified as a strong interaction, yeast growth observable between 3 and 6 days was considered as weaker interaction, while the absence of yeast growth indicated no interaction.

### Promoter analysis

pISV23 binary vector providing kanamycin selection for the bacteria and BASTA herbicide selection for the transgenic plants was used for the construction of promoter-reporter gene fusions. In the case of *AtCDC20.1*, a 750 bp promoter fragment or a 1244 bp region (including the 750 bp promoter as well as the first exon and intron) was fused to the GUS (β-glucuronidase) reporter gene of the vector, while in the case of *AtCDC20.2* the promoter was 1007 bp and the promoter together with the first exon and intron was 1396 bp. Plants were transformed with the “flower dip” method [48]. Minimum ten different lines for each transformation were selected. For GUS staining, the plant material was immersed in the enzymatic reaction mixture (1 mg/ml of 5-bromo-4-chromo-3-indolyl β-d-glucuronide, 2 mM ferricyanide, and 2 mM ferrocyanide in 100 mM phosphate buffer, pH 7.4) and incubated at 37°C in the dark until the coloration was observed (2–16 hours). The plant material was cleared with ethanol washes and examined under a light microscope (Leica).

### Translational fusions

For the cloning of the *AtCDC20.1* and *AtCDC20.2* promoter-ORF-GFP fusions the CaMV 35S promoter was removed from the pB7FWG2 vector [49] with SpeI and SacI digestion and re-ligation of the vector resulting in pB7FWG2Δ35S. The genomic regions (promoter with ORF) were inserted in frame with the GFP coding sequence in pB7FWG2Δ35S. Transgenic plants expressing these translational fusion proteins were obtained with BASTA selection. GFP was visualized by confocal (Leica) and fluorescent (Nikon) microscopy.

### T-DNA mutants and RNA interference lines

The T-DNA insertion lines were obtained from NASC (Nottingham Arabidopsis Stock Center). Insertion mutant information was obtained from the SIGnAL website at “http://signal.salk.edu”. The GK568G01 and GK702F07 lines of the GABI-KAT library [50], the SALK002496, SALK083223, SALK087779, SALK114279C and SALK136724 lines of the SALK library [51] and the SAIL813A03 line of SAIL (Syngenta Arabidopsis Insertion Library) library [52] were used to create homozygous lines. Kanamycin selection was used for SALK lines, sulfadiazine for GABI-KAT lines and BASTA for the SAIL line.

A 117 bp long region of the coding sequence of *AtCDC20.2* was amplified by specific oligos (5′ ctggacaggttcataccg, 5′ ctctttggatggtgaac) and cloned in the pB7GWIWG2(II) binary vector [49] for RNAi experiments. BASTA resistance was used for selection of transformed lines.

### Flow cytometry

Nuclear DNA content was measured at 18 days post germination in the first leaves according to [2], using a Partec CyFlow SL3 cytometer and the FlowMax software (Partec). The ER index was calculated according to [53]. The number of nuclei at each endoploidy level was multiplied by the number of ER cycles necessary to reach the corresponding ploidy level and the sum of the resulting products was divided by the total number of nuclei.

### Phylogenetic and gene structure analysis

Protein sequences were extracted from the PLAZA database [24]. The sequences were aligned by ClustalW2 [54] and presented using the Boxshade software (http://www.ch.embnet.org/). A phylogenetic tree was generated with the MEGA software [55] using the alignment generated by ClustalW2 and the Neighbor-joining method with 1000 bootstraps. Gene structures (intron-exon organization) were extracted from the PLAZA database and individually corrected.

### Histology and microscopy

Root length was measured from the root tip until the root/hypocotyl border. For observation of the root meristem, root morphology and leaf pavement cells, the roots and leaves were stained with FM4-64 (5 µM) and analyzed with a Leica-SP2 confocal microscope (excitation/emission 488/650). Meristem size was measured from the root tip until the first elongating cells. All calculations were made using the ImageJ software (NIH). Statistical calculation for the area of pavement cells was performed with the R software version 2.10.1 (http://www.r-project.org).

### Western blot analysis

Protein extracts were prepared from 50 ml of yeast cultures using the Yeast YPX™ Protein Extraction Kit (Protein Discovery) containing Proteoloc™ Protease Inhibitor Cocktails (Protein Discovery). Samples were analyzed by SDS-PAGE followed by Western Blot using 1∶5000 mouse monoclonal Anti-HA (clone 12CA5, Roche) and 1∶6000 ECL Anti-mouse IgG, peroxidase-linked secondary antibody (NXA931, GE Healthcare).

## Supporting Information

Figure S1 **Alignment of plant CDC20 proteins.** The sequences are annotated by their accession numbers in the PLAZA database. The first two letters indicate the plant species: AL, *Arabidopsis lyrata*; AT, *Arabidopsis thaliana*; PT, *Populus trichocarpa*; CP, *Carica papaya*; GM, *Glycine max*; VV, *Vitis vinifera*; SB, *Sorghum bicolor*; ZM, *Zea mays* and OS, *Oryza sativa*.
(DOC)

Figure S2 **Phylogenetic analysis of plant CDC20 proteins.** The tree was generated using the alignment of Figure S1. Numbers next to the branches indicate the bootstrap values in %. The accession numbers of the proteins are as in Figure S1. The intron-exon organization of each gene is indicated, the conserved exons are in orange and fused exons are in blue, intervening introns are indicated with black lines (not in scale).
(TIF)

Figure S3 **Conservation of exon-intron boundaries in plant CDC20 genes.** The alignment in Figure S1 was used to indicate with colored highlights the exons in the plant *CDC20* genes. Yellow and green exons are conserved with respect to the *AtCDC20*.1 and *AtCDC20.2* genes while exons in blue correspond to fused exons resulting from the loss of one or more introns.
(DOC)

Figure S4 **Production of the Arabidopsis CDC20 isoforms and mitotic cyclins in yeast cells.** The presence of CDC20 and cyclin proteins expressed from the Y2H pGADT7 vector was detected in yeast total protein extracts by Western Blot analysis with the anti-HA antibody. Upper panel, production of the five AtCDC20 proteins as indicated. Lower panel, production of the Arabidopsis cyclin proteins as indicated. Empty corresponds to the analysis of protein extracts of yeast containing the empty pGADT7 vector.
(TIF)
