# Supplementary material for: Conserved CDC20 Cell Cycle Functions Are Carried out by Two of the Five Isoforms in Arabidopsis thaliana
Source: PLoS One. 2011 Jun 8;6(6):e20618. doi: 10.1371/journal.pone.0020618 (PMC3110789; doi:10.1371/journal.pone.0020618)
Supplement: Figure S1 — Alignment of plant CDC20 proteins. The sequences are annotated by their accession numbers in the PLAZA database. The first two letters indicate the plant species: AL, Arabidopsis lyrata; AT, Arabidopsis thaliana; PT, Populus trichocarpa; CP, Carica papaya; GM, Glycine max; VV, Vitis vinifera; SB, Sorghum bicolor; ZM, Zea mays and OS, Oryza sativa. (DOC) [file pone.0020618.s001.doc]

AL4G08930 1 MDSGINTSS-HFKP------------------QARCPLQRNFLPKKTSKENPERFIPNR-
AL5G12940 1 MDSGINTSS-RLNP------------------QARCPLQRNFLPKNTSKENPERFIPNR-
AT5G26900 1 ----MDSG-----M------------------RATCTVPEHFLPRKLSKQNLDRFIPNR-
AT5G27080 1 ----MDS--------------------------DTCTVPDHFLPRKLSKQNLDRFIPNR-
AT5G27570 1 ---MMNTSS-HLKA------------------QASCPLVEHFLRRKLSKENFDRFIPNR-
AL6G28380 1 ----MAQ--------------------------AWFPLEEHFLPRKLSKENLDRFIPNR-
AL7G07950 1 MDAGMN-TSSHYKS------------------QARCPLQEHFLPRKTSKENLDRFIPNR-
AT4G33270 1 MDAGMNNTSSHYKT------------------QARCPLQEHFLPRKPSKENLDRFIPNR-
AT4G33260 1 MDAGLN----------------------------RCPLQEHFLPRKNSKENLDRFIPNR-
PT13G04450 1 MDAGSINSSSSLKA------------------QSRFPLQQQFLPRMNSKENLDRFIPNR-
PT19G03660 1 -----MNTSSSLKA------------------QSRFPLQQQFLPRTNSKENLDRFIPNR-
PT16G11830 1 MDAGSLNSSSYMKA------------------QSRFPLQEQFLHRKNSKDNLDRFIPNR-
CP00039G01100 1 MDVGSGKAYSDLNA------------------QSRWPLH-QFFHRRNSKDNLDRFIPNR-
CP01199G00020 1 MDVGSVKVSSDLKV------------------QSRCPLQDQFLVKRNSKENLDRFIPNR-
GM01G43980 1 MDAGSLSSSGTLKT------------------RSRYPLQEQFIQRKSSKENLDRFIPNR-
GM11G01450 1 MDAGSLSSSGTLKT------------------RSRYPLQEQFIQRKSSKENLDRFIPNR-
VV15G00180 1 MDAGSLTSS--NKY------------------QSKCPDQ-----RRTVRENLDRFIPNR-
GM03G36300 1 MDVG-SWSSSPSKI------------------KSRFSFQDRLFRRKNSQENLDRFIPNR-
GM08G24480 1 MGGDNNVSNPPAKE------------------RKTPAYHYRYRHCKTT--ILDRFIPNR-
VV05G00820 1 MDAGSLN---------------------------SCPQQVQFLQR---WENLDRFIPNR-
VV05G00850 1 MDAGSLN---------------------------SCPQQVQFLQR---WENLDRFIPNRS
VV05G00770 1 MDAGSLN---------------------------SCPQQVQFLQR---WENLDRFIPNR-
VV05G06570 1 MDAGSLN---------------------------SCPQQVQFLQS---WENLDRFIPNR-
VV16G06260 1 MDAGSLSSL-NCNS------------------TYKCPLQEQFHRRRKTRENLDRFIPNR-
SB04G009980 1 MDAGTYSISSEKSHKAAKAAAAPRPPLQEAGSQPYMPSLSTGSRNPSAKCYGDRFIPDR-
ZM05G20580 1 MDAGTYSISSEKSH----TAAAARPPLQEAGTRPYMPSLSTGSRNPSAKCYGDRFIPDR-
OS02G47180 1 MDAGSHSISSEKSH-----GLAPRPPLQEAGSRPYMPSLSTASRNPSAKCYGDRFIPDR-
ZM04G17500 1 MDAGSRSISSAKNR-AAAVAAAPRPPLQEAGSRPYMPSLSSGPRNPSAKCYGDRFIPDR-
OS04G51110 1 MDAGSHSISSEKSS----RYVAPRQPLQEAGSRPYMPSLSTASRNPSAKCYGDRFIPDR-
PT16G06730 1 MDSSSSSTTTRMFH--------------------PRSALRENPQRKKSYENLDRFIPNR-


AL4G08930 41 SAMDFDYAHFQLTEGRNVKDEAT-----KVSSSPSREAYRKQLAETMNLNRTRILAFRNK
AL5G12940 41 SAMDFDYAHFQLTEGRNEKDEAT-----KVRSSPSREAYRKQLAETMNLNRTRILAFRNK
AT5G26900 33 SAKDFDFANYALTQGS--KRNLD-----EVTSAS-RKAYMTQLAVVMNQNRTRILAFRNK
AT5G27080 30 SAMDFDFANYALTQGR--KRNVD-----EITSAS-RKAYMTQLAVVMNQNRTRILAFRNK
AT5G27570 38 SAMDFDFANYALTQGR--KRNVD-----EVTSAS-RKAYMTQLAEAMNQNRTRILAFRNK
AL6G28380 30 SAMDFDYAPYALTEGR--KPKV------EVTSAS-RKAYMNQLAETMNQNRTRILAFRNK
AL7G07950 41 SAMDFDYAHFALTEGRKGNDQTA-----AVSSPS-KEAYRKQLAETMNLNHTRILAFRNK
AT4G33270 42 SAMNFDYAHFALTEGRKGKDQTA-----AVSSPS-KEAYRKQLAETMNLNHTRILAFRNK
AT4G33260 32 SAMNFDYAHFALTEERKGKDQSA-----TVSSPS-KEAYRKQLAETMNLNHTRILAFRNK
PT13G04450 42 SAMDMDYAHFMLTEGRKG-KENP------TVNSPSREAYRKQLAESLNMNRTRILAFKNK
PT19G03660 37 SAMDMDYARFMLTEGRKG-KENP------TVNSPSREAYRKQLADSLNMNRTRILAFKNK
PT16G11830 42 SAMDLDYAHYMLTQGRKGGKENPT----ATVNSPSREAYRKQLAEALNLNRTRILAFKNK
CP00039G01100 41 SAVDMDYARYMLTDG-RKGKENPVREGAVETSSPS-NAYQKQLAEVLNINRTRILAFKNK
CP01199G00020 42 SAMDMDYARFMLTAGGRKGKENPAGGGEAAVSSPSSDAYQKQLAEAFNMNRTRILAFKNK
GM01G43980 42 SAMDFDYAHYMLTEGNKGKENPD-------VCSPSREAYRKQLAESLNMNRTRILAFKNK
GM11G01450 42 SAMDFDYAHYMLTEGNKGKENPD-------VCSPSREAYRKQLAESLNMNRTRILAFKNK
VV15G00180 35 SAMDFDYAHYMLTEGRKGKENPA-------ASSPSKEAYRKQMAETLNINRTRILAFKNK
GM03G36300 41 SAMDFDYAHYMLTEGNKKGKE-KEN---PVVTSPSREAYQKQLAEAFNMNRTRILAFKNK
GM08G24480 40 SAMDFDYAHYMLTEGNKKGKEEKKN---PLVMSPSREAYQKQLADAFNMNRTRILAFKSK
VV05G00820 30 SAMDFDFAHYMLTERGKGKENQSV------VRSQSKEAYLKLLAETFNMNRSRILAFKNK
VV05G00850 31 AMMDFDFAHYMLTERGKGKENQS-------VRSQSKEAYLKLLAETFNMNRSRILAFKNK
VV05G00770 30 SAMDFDFAHYMLTKRGKGKENQSD------VRSQSKEAYLKLLAETFNMNRSRILAFKNK
VV05G06570 30 SAMDFDFAHYMLTKRGKDKENQSV------VRSQSKEAYLKLLAETFNMNRSRILAFKNK
VV16G06260 41 SAMDFDYAHYMLTKGRKGKENPS-------VLSPSIEAYLKLLANTFHMNRGRILAFKNK
SB04G009980 60 SAMDMDVAQYLLTEPRKDKENAAAAAS------PSKEMYRRLLAEKLLNNRTRILAFRNK
ZM05G20580 56 SAMDMDMAHYLLTEPRRDKENAVAAS-------PSKEAYRRLLAEKLLNNRTRILAFRNK
OS02G47180 55 SAMDMDMAHYLLTEPKKDKENAAASP--------SKEVYRRLLAEKLLNNRTRILAFRNK
ZM04G17500 59 SAMDMDLAHYLLTEPRRDKENASGMAA-----SPSKEAYRRLLAEKLLNNRTRILAFRSK
OS04G51110 56 SAMDMDMAHYLLTEPRKDKENAAASP--------AKEAYRKLLAEKILNNRTRILSFRNK
PT16G06730 40 SAMDMDFAHYMLTEGRKAKESPP-----------SQSLYQKLLAEAFNMNGRRILAFKNK

AL4G08930 96 PQTP-VQLLPREHSVYSLYQQPKSVKPRRYIPQNCERALDAPDIVDDFYLNLLDWGSANV
AL5G12940 96 PQAP-VQLLPREHSVYSLYQQPKSVKPRRYIPQNCERALDAPDIVDDFYLNLLDWGSANV
AT5G26900 85 PKS----LLSTNHSD-SPHQNPKPVKPRRYIPQNSERVLDAPGLRDDFSLNLLDWGSANV
AT5G27080 82 PKA----LLSSNHSD-SPHQNPKSVKPRRYIPQNSERVLDAPGLMDDFYLNLLDWGSANV
AT5G27570 90 PKA----LLSSNHSD-PPHQQPISVKPRRYIPQNSERVLDAPGIADDFYLNLLDWGSSNV
AL6G28380 81 PKA----LLSSNHSD-SPHEQSKSVKRRRYIPQNSEKILDAPGIVDDFYLNLLDWGSSNV
AL7G07950 95 PQAP-VELLPSNHSA-SLHQQPKSVKPRRYIPQTSERTLDAPDIVDDFYLNLLDWGSANV
AT4G33270 96 PQAP-VELLPSNHSA-SLHQQPKSVKPRRYIPQTSERTLDAPDIVDDFYLNLLDWGSANV
AT4G33260 86 PQAP-VELLPSNHSA-SLHQQPKSVKPRRYIPQTSERTLDAPDIVDDFYLNLLDWGSANV
PT13G04450 95 PPAP-VELMPQDH--SHHHHQPKTAKPRRHIPQTSERTLDAPDLVDDFYLNLLDWGSSNV
PT19G03660 90 PPAP-VELMPQDH--SHHHHQPKTAKPRRHIPQTSERTLDAPDLVDDFYLNLLDWGSSNV
PT16G11830 98 PPTP-VELIPRDHLSSSLHYQAKPTKPRRYIPQTSERTLDAPDLVDDFYLNLLDWGSKNV
CP00039G01100 99 PTET-SKWIPNEHLVSSLQP-PRSTKPLRHIPQTSEKTLDAPDIVDDFYLNILDWGSANV
CP01199G00020 102 PPAS-SELIPNEHISSSFQP-AKPTKPRRYIPQTSERTLDAPDIVDDFYLNLLDWGSANV
GM01G43980 95 PPAP-VDLIPHEMS--THTHDNKPAKPKRFIPQTSEKTLDAPDLVDDYYLNLLDWGSANV
GM11G01450 95 PPAP-LDLIPHEMS--TYTHDNKPAKPKRFIPQSSEKTLDAPDIVDDYYLNLLDWGSANV
VV15G00180 88 PPTP-VELIPQEFYSASIPQQSKASKPRRHIPQTSERTLDAPDLVDDYYLNLLDWGSSNV
GM03G36300 97 PRTP-VELIPSSILN-PPPPPPNSSKPRRYIPQSSEKTLDAPDILDDYYLNLLDWGSGDV
GM08G24480 97 PRTRRVELIPNSIFS-PPPPP-ISSKHRRHIPQSSERVLDAPDILDDFYLNLLDWGNNNV
VV05G00820 84 PPTP-VKLIPDEFYS--SVHQSKPSKPLRRIPQTPERTLDAPDIIDDFCLNLMDWGSSNV
VV05G00850 84 PPTP-VKLIPDEFYS--SVHQSKPSKPLRRIPQTPVRTLDAPDIIDDFCLNLMDWGSSNV
VV05G00770 84 PPTP-VKLIPDEFYS--SVHQSKPSKPVRRIPQTPERTLDAPNIIDDFCLNLMDWGSSNV
VV05G06570 84 PHTP-VKLIPDEFYS--SVHQSKRSKPLRHIPQTPERTLDAPDIIDDFCLNLMDWSSSNV
VV16G06260 94 PPTP-VELTPREFLS--PVRQFKPSKPKQHIPQTPERTLDAPDIIDDYYLNLLDWGSSNI
SB04G009980 114 PPEP--ENVSATIAASA--HHAKPAKQRRHIPQSAERTLDAPELVDDYYLNLLDWGSNNV
ZM05G20580 109 PPVS--ENVSAAITASS--HHAKLVKQRRHIPQSAERTLDAPELVDDYYLNLLDWGSNNV
OS02G47180 107 PPEP--ENVSAADTAST--HQAKPAKQRRYIPQSAERTLDAPDLVDDYYLNLLDWGSKNV
ZM04G17500 114 PPEP--ENVSFADTTSSN-LQAKPAKQRRHIPQSAERTLDAPELVDDYYLNLLDWGSNNV
OS04G51110 108 PPEP--ESILTELRADAASIQAKPAKQRRYIPQSAERTLDAPELVDDYYLNLLDWGSSNV
PT16G06730 89 PPTL---VDPIPLFSSSSVHSSKPVKPQRHIPQRPEMTLDAPDIVDDFYLNLLDWGNNNV


AL4G08930 155 VAIALGRSVYLWDASSGSVSELVTVDEDMGPVTSINWAQDGLNLAVGLDNSEVQLWDSVA
AL5G12940 155 LAIALGRTVYLWDASNGSVSELVTVDEDMGPVTSINWAQDGLNLAVGLDNSEVQLWDSVA
AT5G26900 140 LAIALGDTVYLWDASSGSTSELVTIDEDKGPVTSINWTQDGLDLAVGLDNSEVQLWDCVS
AT5G27080 137 LAIALGDTVYLWDASSGSTSELVTIDEDKGPVTSINWTQDGLDLAVGLDNSEVQLWDFVS
AT5G27570 145 LAIALGDTVYLWDASSGSTYKLVTIDEEEGPVTSINWTQDGLDLAIGLDNSEVQLWDCVS
AL6G28380 136 LALALGHSIYLRDASSDSTSMLVTIDEEKGPVTSINWMQDGCTLAIGLDNSEVQIWDSAS
AL7G07950 153 LAIALDHTVYLWDASTGSTSELVTIDEEKGPVTSINWAPDGRHVAVGLNNSEVQLWDSAS
AT4G33270 154 LAIALDHTVYLWDASTGSTSELVTIDEEKGPVTSINWAPDGRHVAVGLNNSEVQLWDSAS
AT4G33260 144 LAIALDHTVYLWDASTGSTSELVTIDEEKGPVTSINWAPDGRHVAVGLNNSEVQLWDSAS
PT13G04450 152 LAIALGSTVYLWDASDGSTSELVTVDDEDGPITSVNWAPDGRHIAIGLNNSHIQLWDSAS
PT19G03660 147 LAIALGSTVYLWDASDGSTSELVTVDDEDGPVTSVNWAPDGRHIAIGLNNSHIQLWDSAS
PT16G11830 157 LAIALENTVYLWDASNGSTSELVTVGDEVGPVTSVNWAPDGLHLAIGLNNSNVQLWDSAS
CP00039G01100 157 LAIALGSTVYLWDASTGSASELVTIDDEDGPVTSLSWAPDGRNIAIGLNNSHVQLWDSGS
CP01199G00020 160 LAIALGSTVYLWDASTGSTSELVTVDDEDGPVTSLSWAPDGRHIAVGLNNSRVQLWDSAS
GM01G43980 152 LAIALGSTVYLWDATNGSTSELVTVDDEDGPVTSLSWAPDGRHIAVGLNNSEVQLWDTTS
GM11G01450 152 LAIALGSTVYLWDARNGSTSELVTVDDEDGPVTSVSWAPDGRHIAVGLNNSEVQLWDTSS
VV15G00180 147 LAIALGGTVYLWDASDGSTSELVTLEDETGPVTSVSWAPDGRHIAIGLNNSDVQLWDSTA
GM03G36300 155 LSIALGNTVYLWNASDSSTAELVTVDEEDGPVTSVAWAPDGRHVAIGLNNSHVQLWDSHA
GM08G24480 155 LSIALGNTVYIWDASYSSTAELVTVDEEEGPVTSVAWAPDGCHVAIGLNNSHVLLWDSNV
VV05G00820 141 LALALQNTVYLWDASNGSASELVTVDDENGPVTSVSWAADGQYIAIGLKSSDVQLWDSTA
VV05G00850 141 LALALQNTVYLWDASNGSASELVTVDDENGSVTSVSWAADGQYIAIGLNSSDVQLWDSTA
VV05G00770 141 LALALQNTVYLWDASNGSASELVTVDDENGPVTSVSWAADGQYIAIGLNSSDVQLWDSTT
VV05G06570 141 LALALQNTVYLWDASNGSASELVTVDDENGPVTSVSWAADGQYIAIGLNSSDVQLWDSTA
VV16G06260 151 LAIGLGSTVHFWDGSNGSTSELVTVDDENGPVTSISWAADGQHIAIGLNNSDVQLWDSTA
SB04G009980 170 LSIALGDTVYLWDASTGSTSELVTIDEDSGPITSVSWAPDGKHIAVGLNSSDVQLWDTSS
ZM05G20580 165 LSIALGDTVYLWDASSGSTSELVTIHEDSGPITSVNWAPDGHHIAIGLNSSDIQLWDTSS
OS02G47180 163 LSIALGDTVYLWDASSGSTSELVTVDEDSGPITSVSWAPDGQHVAVGLNSSDIQLWDTSS
ZM04G17500 171 LSIALGDTVYLWDASSGSTSELVTVGEDSGPVTSVSWAPDGRHMAVGLNSSDVQLWDTSS
OS04G51110 166 LSIALGNSVYLWDATNSSTSELVTVDEDNGPVTSVSWAPDGRHIAVGLNSSDVQLWDTSS
PT16G06730 146 LAIALGTTVYLWNASNSSISEVVTVDEEDGPVTSISWAPDGRHLAVGLDNSNVQLWDSAT

AL4G08930 215 SRKVRTLKDGHQS--RVGSLAWNSHILTTGGMDGKIIDNDVRVRSHVVKTYRGHTLEVCG
AL5G12940 215 SRKVRTLKGGHQS--RVGSLAWNNHILTTGGMDGKIINNDVRVRSHVVKTYRGHTLEVCG
AT5G26900 200 NRQVRTLRGGHES--RVGSLAWDNHILTTGGMDGKIVNNDVRIRSSIVETYLGHTEEVCG
AT5G27080 197 NRQVRTLIGGHES--RVGSLAWNNHILTTGGMDGKIVNNDVRIRSSIVGTYLGHTEEVCG
AT5G27570 205 NRQVRTLRGGHES--RVGSLAWNNHILTTGGMDGKIVNNDVRIRSSIVETYLGHTEEVCG
AL6G28380 196 NSQLRTLRGGHQT--RVGSLAWNNHILTTGGRDGKIINNDVRIRSSIVGSYLGHTDEVCG
AL7G07950 213 NRQLRTLKGGHQS--RVGSLAWNNHILTTGGMDGLIINNDVRIRSPIVETYRGHTQEVCG
AT4G33270 214 NRQLRTLKGGHQS--RVGSLAWNNHILTTGGMDGLIINNDVRIRSPIVETYRGHTQEVCG
AT4G33260 204 NRQLRTLKGGHQS--RVGSLAWNNHILTTGGMDGLIINNDVRIRSPIVETYRGHTQEVCG
PT13G04450 212 NRQLRTLKGGHRS--RVGSLAWNNHILTTGGMDGQIINNDVRIRSHIVETYRGHTQEVCG
PT19G03660 207 NRQLRTLKGGHRS--RVGSMAWNNHILTTGGMDGQIINNDVRIRSHIVETYRGHTQEVCG
PT16G11830 217 CKQLRNLRGCHRS--RVGSMAWNNHILTTGGMDGKIINNDVRIRSHIVETYRGHQQEVCG
CP00039G01100 217 NRQLRTLGGGHSHGCRVGSLAWNNHILTTGGMDGQIINNDVRVRSHIVETYRGHRHEVCG
CP01199G00020 220 NRQLRTLRGGHNHGSRVGSLAWNNHILTTGGMDGQIINNDVRVRSHIVETYRGHRQEVCG
GM01G43980 212 NRQLRTLRGGHRQ--RVGSLAWNNHILTTGGMDGRIVNNDVRIRSHVVETYSGHEQEVCG
GM11G01450 212 NRQLRTLRGGHRQ--RVGSLAWNNHILTSGGMDGRIVNNDVRIRSHVVETYSGHEQEVCG
VV15G00180 207 NRLLRTLKGGHAS--RVGSLAWNNHVLTTGGMDGKIINNDVRVRSHIVETYRGHRQEVCG
GM03G36300 215 SRLLRTLKGGHQA--RVGSLSWNNHILTTGGMDGRIVNNDVRVRHHIVESYRGHQQEICG
GM08G24480 215 SRLVRTLRGGHQA--RVGSLSWNNHILTTGGMDGRIVNNDVRVRHHIGESYRGHQQEVCG
VV05G00820 201 NRLLRTLRGGHQS--RVGSLDWKNHILTTGGMDGQIINNDVRVHSHIVATFRGHRQEVCG
VV05G00850 201 NRLLRTLRGGHQS--RVGSLDWKNHILTTGGMDGQIINNDVRAHSHIVATFRGHRQEVCG
VV05G00770 201 NRLLRTLRGGHQS--RVGSLDWKNHILTTGGMDGQIINNDVRVHSHIVATFRGHRQEVCG
VV05G06570 201 NRLLRTLRGGHQS--RVGSLDWKNHILTTGGMDGQIINNDVRVHSHIVATFRGHRQEVCG
VV16G06260 211 NQLLRTLRGGHQS--RVGSLAWNNHILTTGGRDGKIINNDVRVRSHIVETYRGHHQEVCG
SB04G009980 230 NRLLRTLRGVHEA--RVGSLAWNNSILTTGGMDGKIVNNDVRIRNHVVQTYEGHSQEVCG
ZM05G20580 225 NRLLRTLRGVHEE--RVGSLAWNNNILTTGSMDGKIVNNDVRIRNHVVQTYEGHSQEVCG
OS02G47180 223 NRLLRTLRGVHES--RVGSLAWNNNILTTGGMDGNIVNNDVRIRNHVVQTYQGHSQEVCG
ZM04G17500 231 NRLLRTLRGAHEA--RVGSLAWNNSVLTTGCMDGKIVNNDVRIRDHVVQRYEGHSQEVCG
OS04G51110 226 NRLLRTMRGVHDS--RVGSLAWNNNILTTGGMDGKIVNNDVRIRNHVVQTYQGHQQEVCG
PT16G06730 206 NQMLRTLRGGHRL--RVTSLAWNHHLLTTGGKDAKVINNDVRIREHIVESYEGHRQEVCG


AL4G08930 273 LKWSESGQHLASGGNENVVNVWD---------CSTGRSLHRFQEHTSAVKALAWCPFQSG
AL5G12940 273 LKWSESGQHLASGGNDNLVNVWE---------HSTRRSLHRFEEHTSAVKALAWCPFQSG
AT5G26900 258 LKWSESGNKQASGGNDNVVHIWD---RSLASSKQTRQWLHRFEEHTAAVRALAWCPFQAS
AT5G27080 255 LKWSESGKKLASGGNYNVVHIWDH--RSVASSKPTRQWLHRFEEHTAAVRALAWCPFQAT
AT5G27570 263 LKWSESGKKLASGGNDNVVHIWDH--RSVASSNPTRQWLHRFEEHTAAVRALAWCPFQAS
AL6G28380 254 LKWSESGKQLASGGNDKVVHIWD---RSLASSNSTRKWLQRFEGHTAATKALAWCPFQAN
AL7G07950 271 LKWSGSGQQLASGGNDNVVHIWD---RSVASSNSNTQWLHRLEEHTSAVKALAWCPFQAN
AT4G33270 272 LKWSGSGQQLASGGNDNVVHIWD---RSVASSNSTTQWLHRLEEHTSAVKALAWCPFQAN
AT4G33260 262 LKWSGSGQQLASGGNDNVVHIWD---RSVASSNSTTQWLHRLEEHTSAVKALAWCPFQAN
PT13G04450 270 LKWSASGQQLASGGNDNLIHIWDR--STALSNS-ATQWLHRLEDHTSAVKALAWCPFQGN
PT19G03660 265 LKWSASGQQLASGGNDNLIHIWDR--STALSNS-ATQWLHRLEDHTSAVKALAWCPFQGN
PT16G11830 275 LKWSASGQQLASGGNDNIIHIWDR--SVASSNS-ATQWFHRLEEHTSAVKALAWCPFQGN
CP00039G01100 277 LKWSGSGQKLASGGNDNLVHIWDR--SLASSSSERQQWLHRLEEHTSAVKALAWCPFQGN
CP01199G00020 280 LKWSGSGQQLASGGNDNLLHIWDR--SMASSNS-ATQWLHRLEEHTSAVKALAWCPFQGN
GM01G43980 270 LKWSASGSQLASGGNDNLLYIWD---RATASSNSATQWLHRLEDHTSAVKALAWCPFQGN
GM11G01450 270 LKWSASGSQLASGGNDNLLYIWD---RATASSNSATQWLHRLEDHTSAVKALAWCPFQGN
VV15G00180 265 LKWSASGQQLASGGNDNLLHIWD---RSSASSNSPTQWLHRMEDHTAAVKALAWCPFQGN
GM03G36300 273 LRWSPSGQQLASGGNDNVIHIWD---RTMVSSNSPTHWLHRFEEHRAAVKALAWCPFQAN
GM08G24480 273 LRWSPSGQQLASGGNDNVIHIWD---RAMVSSNSPTRWLHRFEEHKAAVRALAWCPFQAN
VV05G00820 259 LKWSTSGQQLASGGNDNLLYIWD---RSMASMHSRSQWLHRLEDHTAAVKALAWCPFQRN
VV05G00850 259 LKWSTSGQQLASGGNDNLLHIWD---RSMASMHSRSQWLHRLEDHTAAVKALAWCPFQRN
VV05G00770 259 LKWSTSGQQLASGGNDNLLYIWD---RSMASMHSRSQWLHRLEDHTAAVKALAWCPFQRN
VV05G06570 259 LKWSTSGQQLASGGNDNLLYIWD---RSMASMHSRSQWLHRLEDHTAAVKALAWCPFQRN
VV16G06260 269 LKWSASGQQLASGGNDNMLYIWD---RSMSSSNSRSQWLHRLEDHTAAVKALAWCPFQSN
SB04G009980 288 LKWSGSGQQLASGGNDNLLHIWDVSMASSMPSAGRNQWLHRLEDHTAAVKALAWCPFQSN
ZM05G20580 283 LKWSGSGQQLASGGNDNLLHIWDVSMASPMSTAGRNQWLHRLEDHMSAVKALAWCPFQSN
OS02G47180 281 LKWSGSGQQLASGGNDNLLHIWDVSMASSVPSAGRNQWLHRLEDHTAAVKALAWCPFQSN
ZM04G17500 289 LKWSGSGQQLASGGNDNLLHIWDVSMASSMPSAGRNQWLHRLEDHMAAVKALAWCPFQSN
OS04G51110 284 LKWSGSGQQLASGGNDNLLHIWDVSMASSMPSAGRTQWLHRLEDHLAAVKALAWCPFQSN
PT16G06730 264 LKWSASGQQLASGGNDNLLFIWD---RFMASSNSPRHWLHKLEDHTAAVKALAWCPFQSN

AL4G08930 324 LLATGGGGEDRTIKFWNTRTGACLNSVDTGSQVCSLIWSNKERELLSSHGFTQNQLTLWK
AL5G12940 324 LLATGGGGEDRTIKFWNTRTGACLNSVDTGSQVCSLIWSKKERELLSSHGFTQNQLTLWK
AT5G26900 315 LLATGGGVGDGKIKFWNTHTGACLNSVETGSQVCSLLWSQSERELLSSHGFTQNQLTLWK
AT5G27080 313 LLATGGGVGDGKIKFWNTHTGACLNSVETGSQVCSLLWSQRERELLSSHGFTQNQLTLWK
AT5G27570 321 LLATGGGVGDGKIKFWNTHTGACLNSVETGSQVCSLLWSKSERELLSSHGFTQNQLTLWK
AL6G28380 311 LLATGGGVGDRTIKFWNTHTGACLNSVETGSQVCSLLWSNKERELLSSHGFTQNQLTLWK
AL7G07950 328 LLATGGGGGDRTIKFWNTHTGACLNSVDTGSQVCSLLWSKNERELLSSHGFTQNQLTLWK
AT4G33270 329 LLATGGGGGDRTIKFWNTHTGACLNSVDTGSQVCSLLWSKNERELLSSHGFTQNQLTLWK
AT4G33260 319 LLATGGGGGDRTIKFWNTHTGACLNSVDTGSQVCSLLWSKNERELLSSHGFTQNQLTLWK
PT13G04450 327 LLASGGGGGDKSIKFWNTHTGACLNSIDTGSQVCSLLWNKNERELLSSHGFTQNQLTVWK
PT19G03660 322 LLASGGGGGDKSIKFWNTHTGACLNSIDTGSQVCSLLWNKNERELLSSHGFTQNQLTVWK
PT16G11830 332 LLASGGGGGDRSIKFWNTHTGACLNSIDTGSQVCALLWNKNERELLSSHGFTQNQLVLWK
CP00039G01100 335 LLASGGGEGDRCIRFWNTQTAACLNTVDTGSQVSSLLWNKKERELLSSHGFFHNQLTLWK
CP01199G00020 337 LLASGGGGGDRCIKFWNTHTGACLNTVDTGSQVCALLWNKNERELLSSHGFTHNQLTLWK
GM01G43980 327 LLASGGGSGDRCIKFWNTHTGACLNSIDTGSQVCSLLWNKNERELLSSHGFTQNQLTLWK
GM11G01450 327 LLASGGGSGDRCIKFWNTHTGACLNSIDTGSQVCSLLWNKNERELLSSHGFTQNQLTLWK
VV15G00180 322 LLASGGGGGDRCIKFWNTHTGACLNSVDTGSQVCALLWNKNERELLSSHGFTQNQLTLWK
GM03G36300 330 LLASGGGGGDHCIKFWNTHTGACLNSVDTGSQVCALLWSKNERELLSSHGFTQNQLALWK
GM08G24480 330 LLASGGGGGDHCIKFWNTHTGACLNSVDTGSQVCALVWNKNERELLSSHGFTQNQLALWK
VV05G00820 316 LLASGGGGSDGCIKFWNTHTSACLNSVDTGSQVCALLWNKNERELLSSHGFMQNQMTLWM
VV05G00850 316 LLASGGGGSDGCIKFWNTHTGACLNSVDTGSQVCALLWNKNERELLSSHGFMQNQMTLWM
VV05G00770 316 LLASGGGGSDSCIKFWNTHTGACLNSVDTGSQVCALLWNKNERELLSSHGFMQNQMTLWM
VV05G06570 316 LLASGGGGSDCCIKFWNTHTGACLNSVDTGSQVCALLWNKNERELLSSHGFMQNQLTLWM
VV16G06260 326 LLASGGGGNDLCIRFWNTHTGACLNTVDTGSQVCALLWNKKERELLSSHGFSQNQLTLWK
SB04G009980 348 LLATGGGGSDRCIKFWNTHTGACLNSVDTGSQVCALLWNKNERELLSSHGFTQNQLTLWK
ZM05G20580 343 LLATGGGGSDRCIKFWNTHTGACLNSVNTGSQVCALLWNKNERELLSSHGFTQNQLTLWK
OS02G47180 341 LLATGGGGSDRCIKFWNTHTGACLNSVDTGSQVCALLWNKNERELLSSHGFTQNQLTLWK
ZM04G17500 349 LLATGGGGSDRCIKFWNTHTGVCLNSVDTGSQVCALLWNKNERELLSSHGFTQNQLTLWK
OS04G51110 344 LLASGGGGSDRCIKFWNTHTGACLNSIDTGSQVCSLVWNKNERELLSSHGFAQNQLTLWK
PT16G06730 321 LLASGGGGNDRHIKFWNTQTGTCLNSVDTGSQVCALQWNKHERELLSSHGFTENQLILWK


AL4G08930 384 YPSMVKMAELNGHTSRVLYMSQSPDGCTVASAAGDETLRLWNVFGIPE--DAKKAAPKA-
AL5G12940 384 YPSMVKMAELNGHTSRVLYMSQSPDGCTVASAAGDETLRLWNVFGVPE--DAKKAAPKA-
AT5G26900 375 YPSMSKMAELNGHTSRVLFMAQSPNGCTVASAAGDENLRLWNVFGEPP-KTTKKAASKK-
AT5G27080 373 YPSMSKMAELNGHTSRVLFMAQSPNGCTVASAAGDENLRLWNVFGEPP-KTTKKAASKN-
AT5G27570 381 YPSMVKMAELNGHTSRVLFMAQSPDGCTVASAAGDETLRLWNVFGEPP-KTTKKAASKK-
AL6G28380 371 YPSMLKIAELNGHTSRVLYMAQSPDGCTVASAAGDETLRLWNVFGVPPPKTTKKAAPKA-
AL7G07950 388 YPSMVKMAELTGHTSRVLYMAQSPDGCTVASAAGDETLRFWNVFGVPE--TAKKAAPKA-
AT4G33270 389 YPSMVKMAELTGHTSRVLYMAQSPDGCTVASAAGDETLRFWNVFGVPE--TAKKAAPKA-
AT4G33260 379 YPSMVKMAELTGHTSRVLYMAQSPDGCTVASAAGDETLRFWNVFGVPE--TAKKAAPKA-
PT13G04450 387 YPSMVKMAELTGHTSRVLYMAQSPDGCTVATAAGDETLRFWNVFGVPE--VAAKAAPKA-
PT19G03660 382 YPSMVKMAELTGHTSRVLYMAQSPDGCTVATAAGDETLRFWNVFGVPE--IAAKAAPKA-
PT16G11830 392 YPSMLKMAELTGHTSRVLYMAQSPDGCTVATAAGDETLRFWNVFGVPE--VA-KAAPKA-
CP00039G01100 395 YPSMMKVAELTGHKSRVLCMAQSPDGCTVASAAGDERVKLWNVFGVPE--KAAKAARKQ-
CP01199G00020 397 YPSMVKIAELTGHTSRVLYMAQSPDGCTVASAAGDETLRFWNVFGVPE--TAAKAAPKQ-
GM01G43980 387 YPSMVKMAELTGHTSRVLFMAQSPDGCTVASAAADETLRFWNVFGAPE--AASKAAPKA-
GM11G01450 387 YPSMVKMAELNGHTSRVLFMAQSPDGCTVASAAADETLRFWNVFGAPE--AASKAAPKA-
VV15G00180 382 YPSMVKMAELTGHTSRVLFMAQSPDGCTVASAAGDETLRFWNVFGTPE--VAAKPAPKA-
GM03G36300 390 YPSMLKMAELKGHTSRVLYMAQSPNGCTVASAAGDETLRFWNVFGTAQ---ASKPAPTA-
GM08G24480 390 YPSMLKKAELKGHTSRVLYMAQSPNGCTVASAAGDETLRFWNVFGTPQ---ASKPAPKT-
VV05G00820 376 YPSMVKIAELTGHTSRVLFMAQSPDGRTVATAAGDETLKFWNAFG-TP--EVKKAAPKAE
VV05G00850 376 YPSMVKIAELTGHTSRVLFMAQSPDGRIVATAAGDETLKFWNAFG-TP--EVKKAAPKAE
VV05G00770 376 YPSMVKIAELTGHTSRVLFMAQSPDGRTVATAAGDETLKFWNAFG-TP--EVKKASPKAE
VV05G06570 376 YPSMVKTAELTGHTSRVLFMAQSPDGRTVATAAGDETLKFWNAFG-MP--EVKKAAPKAE
VV16G06260 386 YPSMVKITELTGHTSRVLFMAQSPDGCTVVTAAGDETLKFWNVFGTTP--EVKNAAPK--
SB04G009980 408 YPSMVKMAELTGHTSRVLFMAQSPDGCTVASAAADETLRFWNVFGAPE---APKP-VKAS
ZM05G20580 403 YPSMVKMAELSGHTSRVLFMAQSPDGCTVASAAADETLRFWNVFGDPE---VAKPAAKAS
OS02G47180 401 YPSMVKMAELTGHTSRVLFMAQSPDGCTVASAAADETLRFWNVFGSPE---APKPAAKAS
ZM04G17500 409 YPSMVKMAELNGHTSRVLFMAQSPDGCTVASAAADETLRFWNVFGTPE---TPKPAAKAS
OS04G51110 404 YPSMVKMAELTGHTSRVLFTAQSPDGLTVASAAADETLRFWNVFGAPE---APKTATKGS
PT16G06730 381 YPSMVKMAELSGHTSPVLFMTQSPDGYTVASAAGDETLRFWNVFGNPK---AAKPAPKA-

AL4G08930 441 VPQPFSNVN--RIR
AL5G12940 441 VPQPFSNVN--RIR
AT5G26900 433 YPELFSHVN--SLR
AT5G27080 431 YLELFSHVN--SLR
AT5G27570 439 YTDPFAHVN--HIR
AL6G28380 430 YLQIFSHVN--CIR
AL7G07950 445 VSEPFSHVN--RIR
AT4G33270 446 VSEPFSHVN--RIR
AT4G33260 436 VAEPFSHVN--RIR
PT13G04450 444 NPEPFSHLN--RLR
PT19G03660 439 NPEPFSHLN--RIR
PT16G11830 448 NPEPFSRFN--RIR
CP00039G01100 452 NREPFSHLS--RIR
CP01199G00020 454 NPEPFSHLN--RIR
GM01G43980 444 RAEPFSNVN--RIR
GM11G01450 444 RAEPFSNVN--RIR
VV15G00180 439 HPEPFAHLN--RIR
GM03G36300 446 STDPFAHVN--RIR
GM08G24480 446 NVEPFANVN--CIR
VV05G00820 433 HPGPFPHLR--RIR
VV05G00850 433 HPGPFPHLR--RIR
VV05G00770 433 HPGPFPHIR--RIR
VV05G06570 433 HPGPFPHIR--RIR
VV16G06260 442 --ELFPHFS--RIR
SB04G009980 464 HTGMFNSFN--HIR
ZM05G20580 460 HTGMFNSFN--HIR
OS02G47180 458 HTGMFNSFN--HLR
ZM04G17500 466 HTGMFNSFK--HIR
OS04G51110 461 HTGMFNNSNHIHIR
PT16G06730 437 IAEPFANVS--HFR
