# Supplementary material for: Conserved CDC20 Cell Cycle Functions Are Carried out by Two of the Five Isoforms in Arabidopsis thaliana
Source: PLoS One. 2011 Jun 8;6(6):e20618. doi: 10.1371/journal.pone.0020618 (PMC3110789; doi:10.1371/journal.pone.0020618)
Supplement: Figure S3 — Conservation of exon-intron boundaries in plant CDC20 genes. The alignment in Figure S1 was used to indicate with colored highlights the exons in the plant CDC20 genes. Yellow and green exons are conserved with respect to the AtCDC20.1 and AtCDC20.2 genes while exons in blue correspond to fused exons resulting from the loss of one or more introns. (DOC) [file pone.0020618.s003.doc]

AL4G08930 MDSGINTSS-HFKP------------------QARCPLQRNFLPKKTSKENPERFIPNR- 40

AL5G12940 MDSGINTSS-RLNP------------------QARCPLQRNFLPKNTSKENPERFIPNR- 40

AT5G26900 ----MDSG-----M------------------RATCTVPEHFLPRKLSKQNLDRFIPNR- 32

AT5G27080 ----MDS--------------------------DTCTVPDHFLPRKLSKQNLDRFIPNR- 29

AT5G27570 ---MMNTSS-HLKA------------------QASCPLVEHFLRRKLSKENFDRFIPNR- 37

AL6G28380 ----MAQ--------------------------AWFPLEEHFLPRKLSKENLDRFIPNR- 29

AL7G07950 MDAGMN-TSSHYKS------------------QARCPLQEHFLPRKTSKENLDRFIPNR- 40

AT4G33270 MDAGMNNTSSHYKT------------------QARCPLQEHFLPRKPSKENLDRFIPNR- 41

AT4G33260 MDAGLN----------------------------RCPLQEHFLPRKNSKENLDRFIPNR- 31

PT13G04450 MDAGSINSSSSLKA------------------QSRFPLQQQFLPRMNSKENLDRFIPNR- 41

PT19G03660 -----MNTSSSLKA------------------QSRFPLQQQFLPRTNSKENLDRFIPNR- 36

PT16G11830 MDAGSLNSSSYMKA------------------QSRFPLQEQFLHRKNSKDNLDRFIPNR- 41

CP00039G01100 MDVGSGKAYSDLNA------------------QSRWPLH-QFFHRRNSKDNLDRFIPNR- 40

CP01199G00020 MDVGSVKVSSDLKV------------------QSRCPLQDQFLVKRNSKENLDRFIPNR- 41

GM01G43980 MDAGSLSSSGTLKT------------------RSRYPLQEQFIQRKSSKENLDRFIPNR- 41

GM11G01450 MDAGSLSSSGTLKT------------------RSRYPLQEQFIQRKSSKENLDRFIPNR- 41

VV15G00180 MDAGSLTSS--NKY------------------QSKCPDQ-----RRTVRENLDRFIPNR- 34

GM03G36300 MDVG-SWSSSPSKI------------------KSRFSFQDRLFRRKNSQENLDRFIPNR- 40

GM08G24480 MGGDNNVSNPPAKE------------------RKTPAYHYRYRHCKTT--ILDRFIPNR- 39

VV05G00820 MDAGSLN---------------------------SCPQQVQFLQR---WENLDRFIPNR- 29

VV05G00850 MDAGSLN---------------------------SCPQQVQFLQR---WENLDRFIPNRS 30

VV05G00770 MDAGSLN---------------------------SCPQQVQFLQR---WENLDRFIPNR- 29

VV05G06570 MDAGSLN---------------------------SCPQQVQFLQS---WENLDRFIPNR- 29

VV16G06260 MDAGSLSSL-NCNS------------------TYKCPLQEQFHRRRKTRENLDRFIPNR- 40

SB04G009980 MDAGTYSISSEKSHKAAKAAAAPRPPLQEAGSQPYMPSLSTGSRNPSAKCYGDRFIPDR- 59

ZM05G20580 MDAGTYSISSEKSH----TAAAARPPLQEAGTRPYMPSLSTGSRNPSAKCYGDRFIPDR- 55

OS02G47180 MDAGSHSISSEKSH-----GLAPRPPLQEAGSRPYMPSLSTASRNPSAKCYGDRFIPDR- 54

ZM04G17500 MDAGSRSISSAKNR-AAAVAAAPRPPLQEAGSRPYMPSLSSGPRNPSAKCYGDRFIPDR- 58

OS04G51110 MDAGSHSISSEKSS----RYVAPRQPLQEAGSRPYMPSLSTASRNPSAKCYGDRFIPDR- 55

PT16G06730 MDSSSSSTTTRMFH--------------------PRSALRENPQRKKSYENLDRFIPNR- 39

. :****:*

AL4G08930 SAMDFDYAHFQLTEGRNVKDEAT-----KVSSSPSREAYRKQLAETMNLNRTRILAFRNK 95

AL5G12940 SAMDFDYAHFQLTEGRNEKDEAT-----KVRSSPSREAYRKQLAETMNLNRTRILAFRNK 95

AT5G26900 SAKDFDFANYALTQGS--KRNLD-----EVTSAS-RKAYMTQLAVVMNQNRTRILAFRNK 84

AT5G27080 SAMDFDFANYALTQGR--KRNVD-----EITSAS-RKAYMTQLAVVMNQNRTRILAFRNK 81

AT5G27570 SAMDFDFANYALTQGR--KRNVD-----EVTSAS-RKAYMTQLAEAMNQNRTRILAFRNK 89

AL6G28380 SAMDFDYAPYALTEGR--KPKV------EVTSAS-RKAYMNQLAETMNQNRTRILAFRNK 80

AL7G07950 SAMDFDYAHFALTEGRKGNDQTA-----AVSSPS-KEAYRKQLAETMNLNHTRILAFRNK 94

AT4G33270 SAMNFDYAHFALTEGRKGKDQTA-----AVSSPS-KEAYRKQLAETMNLNHTRILAFRNK 95

AT4G33260 SAMNFDYAHFALTEERKGKDQSA-----TVSSPS-KEAYRKQLAETMNLNHTRILAFRNK 85

PT13G04450 SAMDMDYAHFMLTEGRKG-KENP------TVNSPSREAYRKQLAESLNMNRTRILAFKNK 94

PT19G03660 SAMDMDYARFMLTEGRKG-KENP------TVNSPSREAYRKQLADSLNMNRTRILAFKNK 89

PT16G11830 SAMDLDYAHYMLTQGRKGGKENPT----ATVNSPSREAYRKQLAEALNLNRTRILAFKNK 97

CP00039G01100 SAVDMDYARYMLTDG-RKGKENPVREGAVETSSPS-NAYQKQLAEVLNINRTRILAFKNK 98

CP01199G00020 SAMDMDYARFMLTAGGRKGKENPAGGGEAAVSSPSSDAYQKQLAEAFNMNRTRILAFKNK 101

GM01G43980 SAMDFDYAHYMLTEGNKGKENPD-------VCSPSREAYRKQLAESLNMNRTRILAFKNK 94

GM11G01450 SAMDFDYAHYMLTEGNKGKENPD-------VCSPSREAYRKQLAESLNMNRTRILAFKNK 94

VV15G00180 SAMDFDYAHYMLTEGRKGKENPA-------ASSPSKEAYRKQMAETLNINRTRILAFKNK 87

GM03G36300 SAMDFDYAHYMLTEGNKKGKE-KEN---PVVTSPSREAYQKQLAEAFNMNRTRILAFKNK 96

GM08G24480 SAMDFDYAHYMLTEGNKKGKEEKKN---PLVMSPSREAYQKQLADAFNMNRTRILAFKSK 96

VV05G00820 SAMDFDFAHYMLTERGKGKENQSV------VRSQSKEAYLKLLAETFNMNRSRILAFKNK 83

VV05G00850 AMMDFDFAHYMLTERGKGKENQS-------VRSQSKEAYLKLLAETFNMNRSRILAFKNK 83

VV05G00770 SAMDFDFAHYMLTKRGKGKENQSD------VRSQSKEAYLKLLAETFNMNRSRILAFKNK 83

VV05G06570 SAMDFDFAHYMLTKRGKDKENQSV------VRSQSKEAYLKLLAETFNMNRSRILAFKNK 83

VV16G06260 SAMDFDYAHYMLTKGRKGKENPS-------VLSPSIEAYLKLLANTFHMNRGRILAFKNK 93

SB04G009980 SAMDMDVAQYLLTEPRKDKENAAAAAS------PSKEMYRRLLAEKLLNNRTRILAFRNK 113

ZM05G20580 SAMDMDMAHYLLTEPRRDKENAVAAS-------PSKEAYRRLLAEKLLNNRTRILAFRNK 108

OS02G47180 SAMDMDMAHYLLTEPKKDKENAAASP--------SKEVYRRLLAEKLLNNRTRILAFRNK 106

ZM04G17500 SAMDMDLAHYLLTEPRRDKENASGMAA-----SPSKEAYRRLLAEKLLNNRTRILAFRSK 113

OS04G51110 SAMDMDMAHYLLTEPRKDKENAAASP--------AKEAYRKLLAEKILNNRTRILSFRNK 107

PT16G06730 SAMDMDFAHYMLTEGRKAKESPP-----------SQSLYQKLLAEAFNMNGRRILAFKNK 88

: ::* * : ** . . * :* : * ***:*:.*

AL4G08930 PQTP-VQLLPREHSVYSLYQQPKSVKPRRYIPQNCERALDAPDIVDDFYLNLLDWGSANV 154

AL5G12940 PQAP-VQLLPREHSVYSLYQQPKSVKPRRYIPQNCERALDAPDIVDDFYLNLLDWGSANV 154

AT5G26900 PKS----LLSTNHSD-SPHQNPKPVKPRRYIPQNSERVLDAPGLRDDFSLNLLDWGSANV 139

AT5G27080 PKA----LLSSNHSD-SPHQNPKSVKPRRYIPQNSERVLDAPGLMDDFYLNLLDWGSANV 136

AT5G27570 PKA----LLSSNHSD-PPHQQPISVKPRRYIPQNSERVLDAPGIADDFYLNLLDWGSSNV 144

AL6G28380 PKA----LLSSNHSD-SPHEQSKSVKRRRYIPQNSEKILDAPGIVDDFYLNLLDWGSSNV 135

AL7G07950 PQAP-VELLPSNHSA-SLHQQPKSVKPRRYIPQTSERTLDAPDIVDDFYLNLLDWGSANV 152

AT4G33270 PQAP-VELLPSNHSA-SLHQQPKSVKPRRYIPQTSERTLDAPDIVDDFYLNLLDWGSANV 153

AT4G33260 PQAP-VELLPSNHSA-SLHQQPKSVKPRRYIPQTSERTLDAPDIVDDFYLNLLDWGSANV 143

PT13G04450 PPAP-VELMPQDH--SHHHHQPKTAKPRRHIPQTSERTLDAPDLVDDFYLNLLDWGSSNV 151

PT19G03660 PPAP-VELMPQDH--SHHHHQPKTAKPRRHIPQTSERTLDAPDLVDDFYLNLLDWGSSNV 146

PT16G11830 PPTP-VELIPRDHLSSSLHYQAKPTKPRRYIPQTSERTLDAPDLVDDFYLNLLDWGSKNV 156

CP00039G01100 PTET-SKWIPNEHLVSSLQP-PRSTKPLRHIPQTSEKTLDAPDIVDDFYLNILDWGSANV 156

CP01199G00020 PPAS-SELIPNEHISSSFQP-AKPTKPRRYIPQTSERTLDAPDIVDDFYLNLLDWGSANV 159

GM01G43980 PPAP-VDLIPHEMS--THTHDNKPAKPKRFIPQTSEKTLDAPDLVDDYYLNLLDWGSANV 151

GM11G01450 PPAP-LDLIPHEMS--TYTHDNKPAKPKRFIPQSSEKTLDAPDIVDDYYLNLLDWGSANV 151

VV15G00180 PPTP-VELIPQEFYSASIPQQSKASKPRRHIPQTSERTLDAPDLVDDYYLNLLDWGSSNV 146

GM03G36300 PRTP-VELIPSSILN-PPPPPPNSSKPRRYIPQSSEKTLDAPDILDDYYLNLLDWGSGDV 154

GM08G24480 PRTRRVELIPNSIFS-PPPPP-ISSKHRRHIPQSSERVLDAPDILDDFYLNLLDWGNNNV 154

VV05G00820 PPTP-VKLIPDEFYS--SVHQSKPSKPLRRIPQTPERTLDAPDIIDDFCLNLMDWGSSNV 140

VV05G00850 PPTP-VKLIPDEFYS--SVHQSKPSKPLRRIPQTPVRTLDAPDIIDDFCLNLMDWGSSNV 140

VV05G00770 PPTP-VKLIPDEFYS--SVHQSKPSKPVRRIPQTPERTLDAPNIIDDFCLNLMDWGSSNV 140

VV05G06570 PHTP-VKLIPDEFYS--SVHQSKRSKPLRHIPQTPERTLDAPDIIDDFCLNLMDWSSSNV 140

VV16G06260 PPTP-VELTPREFLS--PVRQFKPSKPKQHIPQTPERTLDAPDIIDDYYLNLLDWGSSNI 150

SB04G009980 PPEP--ENVSATIAASA--HHAKPAKQRRHIPQSAERTLDAPELVDDYYLNLLDWGSNNV 169

ZM05G20580 PPVS--ENVSAAITASS--HHAKLVKQRRHIPQSAERTLDAPELVDDYYLNLLDWGSNNV 164

OS02G47180 PPEP--ENVSAADTAST--HQAKPAKQRRYIPQSAERTLDAPDLVDDYYLNLLDWGSKNV 162

ZM04G17500 PPEP--ENVSFADTTSSN-LQAKPAKQRRHIPQSAERTLDAPELVDDYYLNLLDWGSNNV 170

OS04G51110 PPEP--ESILTELRADAASIQAKPAKQRRYIPQSAERTLDAPELVDDYYLNLLDWGSSNV 165

PT16G06730 PPTL---VDPIPLFSSSSVHSSKPVKPQRHIPQRPEMTLDAPDIVDDFYLNLLDWGNNNV 145

* * : *** **** : **: **::**.. ::

AL4G08930 VAIALGRSVYLWDASSGSVSELVTVDEDMGPVTSINWAQDGLNLAVGLDNSEVQLWDSVA 214

AL5G12940 LAIALGRTVYLWDASNGSVSELVTVDEDMGPVTSINWAQDGLNLAVGLDNSEVQLWDSVA 214

AT5G26900 LAIALGDTVYLWDASSGSTSELVTIDEDKGPVTSINWTQDGLDLAVGLDNSEVQLWDCVS 199

AT5G27080 LAIALGDTVYLWDASSGSTSELVTIDEDKGPVTSINWTQDGLDLAVGLDNSEVQLWDFVS 196

AT5G27570 LAIALGDTVYLWDASSGSTYKLVTIDEEEGPVTSINWTQDGLDLAIGLDNSEVQLWDCVS 204

AL6G28380 LALALGHSIYLRDASSDSTSMLVTIDEEKGPVTSINWMQDGCTLAIGLDNSEVQIWDSAS 195

AL7G07950 LAIALDHTVYLWDASTGSTSELVTIDEEKGPVTSINWAPDGRHVAVGLNNSEVQLWDSAS 212

AT4G33270 LAIALDHTVYLWDASTGSTSELVTIDEEKGPVTSINWAPDGRHVAVGLNNSEVQLWDSAS 213

AT4G33260 LAIALDHTVYLWDASTGSTSELVTIDEEKGPVTSINWAPDGRHVAVGLNNSEVQLWDSAS 203

PT13G04450 LAIALGSTVYLWDASDGSTSELVTVDDEDGPITSVNWAPDGRHIAIGLNNSHIQLWDSAS 211

PT19G03660 LAIALGSTVYLWDASDGSTSELVTVDDEDGPVTSVNWAPDGRHIAIGLNNSHIQLWDSAS 206

PT16G11830 LAIALENTVYLWDASNGSTSELVTVGDEVGPVTSVNWAPDGLHLAIGLNNSNVQLWDSAS 216

CP00039G01100 LAIALGSTVYLWDASTGSASELVTIDDEDGPVTSLSWAPDGRNIAIGLNNSHVQLWDSGS 216

CP01199G00020 LAIALGSTVYLWDASTGSTSELVTVDDEDGPVTSLSWAPDGRHIAVGLNNSRVQLWDSAS 219

GM01G43980 LAIALGSTVYLWDATNGSTSELVTVDDEDGPVTSLSWAPDGRHIAVGLNNSEVQLWDTTS 211

GM11G01450 LAIALGSTVYLWDARNGSTSELVTVDDEDGPVTSVSWAPDGRHIAVGLNNSEVQLWDTSS 211

VV15G00180 LAIALGGTVYLWDASDGSTSELVTLEDETGPVTSVSWAPDGRHIAIGLNNSDVQLWDSTA 206

GM03G36300 LSIALGNTVYLWNASDSSTAELVTVDEEDGPVTSVAWAPDGRHVAIGLNNSHVQLWDSHA 214

GM08G24480 LSIALGNTVYIWDASYSSTAELVTVDEEEGPVTSVAWAPDGCHVAIGLNNSHVLLWDSNV 214

VV05G00820 LALALQNTVYLWDASNGSASELVTVDDENGPVTSVSWAADGQYIAIGLKSSDVQLWDSTA 200

VV05G00850 LALALQNTVYLWDASNGSASELVTVDDENGSVTSVSWAADGQYIAIGLNSSDVQLWDSTA 200

VV05G00770 LALALQNTVYLWDASNGSASELVTVDDENGPVTSVSWAADGQYIAIGLNSSDVQLWDSTT 200

VV05G06570 LALALQNTVYLWDASNGSASELVTVDDENGPVTSVSWAADGQYIAIGLNSSDVQLWDSTA 200

VV16G06260 LAIGLGSTVHFWDGSNGSTSELVTVDDENGPVTSISWAADGQHIAIGLNNSDVQLWDSTA 210

SB04G009980 LSIALGDTVYLWDASTGSTSELVTIDEDSGPITSVSWAPDGKHIAVGLNSSDVQLWDTSS 229

ZM05G20580 LSIALGDTVYLWDASSGSTSELVTIHEDSGPITSVNWAPDGHHIAIGLNSSDIQLWDTSS 224

OS02G47180 LSIALGDTVYLWDASSGSTSELVTVDEDSGPITSVSWAPDGQHVAVGLNSSDIQLWDTSS 222

ZM04G17500 LSIALGDTVYLWDASSGSTSELVTVGEDSGPVTSVSWAPDGRHMAVGLNSSDVQLWDTSS 230

OS04G51110 LSIALGNSVYLWDATNSSTSELVTVDEDNGPVTSVSWAPDGRHIAVGLNSSDVQLWDTSS 225

PT16G06730 LAIALGTTVYLWNASNSSISEVVTVDEEDGPVTSISWAPDGRHLAVGLDNSNVQLWDSAT 205

:::.* :::: :. .* :**: :: *.:**: * ** :*:**..* : :**

AL4G08930 SRKVRTLKDGHQS--RVGSLAWNSHILTTGGMDGKIIDNDVRVRSHVVKTYRGHTLEVCG 272

AL5G12940 SRKVRTLKGGHQS--RVGSLAWNNHILTTGGMDGKIINNDVRVRSHVVKTYRGHTLEVCG 272

AT5G26900 NRQVRTLRGGHES--RVGSLAWDNHILTTGGMDGKIVNNDVRIRSSIVETYLGHTEEVCG 257

AT5G27080 NRQVRTLIGGHES--RVGSLAWNNHILTTGGMDGKIVNNDVRIRSSIVGTYLGHTEEVCG 254

AT5G27570 NRQVRTLRGGHES--RVGSLAWNNHILTTGGMDGKIVNNDVRIRSSIVETYLGHTEEVCG 262

AL6G28380 NSQLRTLRGGHQT--RVGSLAWNNHILTTGGRDGKIINNDVRIRSSIVGSYLGHTDEVCG 253

AL7G07950 NRQLRTLKGGHQS--RVGSLAWNNHILTTGGMDGLIINNDVRIRSPIVETYRGHTQEVCG 270

AT4G33270 NRQLRTLKGGHQS--RVGSLAWNNHILTTGGMDGLIINNDVRIRSPIVETYRGHTQEVCG 271

AT4G33260 NRQLRTLKGGHQS--RVGSLAWNNHILTTGGMDGLIINNDVRIRSPIVETYRGHTQEVCG 261

PT13G04450 NRQLRTLKGGHRS--RVGSLAWNNHILTTGGMDGQIINNDVRIRSHIVETYRGHTQEVCG 269

PT19G03660 NRQLRTLKGGHRS--RVGSMAWNNHILTTGGMDGQIINNDVRIRSHIVETYRGHTQEVCG 264

PT16G11830 CKQLRNLRGCHRS--RVGSMAWNNHILTTGGMDGKIINNDVRIRSHIVETYRGHQQEVCG 274

CP00039G01100 NRQLRTLGGGHSHGCRVGSLAWNNHILTTGGMDGQIINNDVRVRSHIVETYRGHRHEVCG 276

CP01199G00020 NRQLRTLRGGHNHGSRVGSLAWNNHILTTGGMDGQIINNDVRVRSHIVETYRGHRQEVCG 279

GM01G43980 NRQLRTLRGGHRQ--RVGSLAWNNHILTTGGMDGRIVNNDVRIRSHVVETYSGHEQEVCG 269

GM11G01450 NRQLRTLRGGHRQ--RVGSLAWNNHILTSGGMDGRIVNNDVRIRSHVVETYSGHEQEVCG 269

VV15G00180 NRLLRTLKGGHAS--RVGSLAWNNHVLTTGGMDGKIINNDVRVRSHIVETYRGHRQEVCG 264

GM03G36300 SRLLRTLKGGHQA--RVGSLSWNNHILTTGGMDGRIVNNDVRVRHHIVESYRGHQQEICG 272

GM08G24480 SRLVRTLRGGHQA--RVGSLSWNNHILTTGGMDGRIVNNDVRVRHHIGESYRGHQQEVCG 272

VV05G00820 NRLLRTLRGGHQS--RVGSLDWKNHILTTGGMDGQIINNDVRVHSHIVATFRGHRQEVCG 258

VV05G00850 NRLLRTLRGGHQS--RVGSLDWKNHILTTGGMDGQIINNDVRAHSHIVATFRGHRQEVCG 258

VV05G00770 NRLLRTLRGGHQS--RVGSLDWKNHILTTGGMDGQIINNDVRVHSHIVATFRGHRQEVCG 258

VV05G06570 NRLLRTLRGGHQS--RVGSLDWKNHILTTGGMDGQIINNDVRVHSHIVATFRGHRQEVCG 258

VV16G06260 NQLLRTLRGGHQS--RVGSLAWNNHILTTGGRDGKIINNDVRVRSHIVETYRGHHQEVCG 268

SB04G009980 NRLLRTLRGVHEA--RVGSLAWNNSILTTGGMDGKIVNNDVRIRNHVVQTYEGHSQEVCG 287

ZM05G20580 NRLLRTLRGVHEE--RVGSLAWNNNILTTGSMDGKIVNNDVRIRNHVVQTYEGHSQEVCG 282

OS02G47180 NRLLRTLRGVHES--RVGSLAWNNNILTTGGMDGNIVNNDVRIRNHVVQTYQGHSQEVCG 280

ZM04G17500 NRLLRTLRGAHEA--RVGSLAWNNSVLTTGCMDGKIVNNDVRIRDHVVQRYEGHSQEVCG 288

OS04G51110 NRLLRTMRGVHDS--RVGSLAWNNNILTTGGMDGKIVNNDVRIRNHVVQTYQGHQQEVCG 283

PT16G06730 NQMLRTLRGGHRL--RVTSLAWNHHLLTTGGKDAKVINNDVRIREHIVESYEGHRQEVCG 263

:*.: . * ** *: *. :**:* *. :::**** : : : ** *:**

AL4G08930 LKWSESGQHLASGGNENVVNVWD---------CSTGRSLHRFQEHTSAVKALAWCPFQSG 323

AL5G12940 LKWSESGQHLASGGNDNLVNVWE---------HSTRRSLHRFEEHTSAVKALAWCPFQSG 323

AT5G26900 LKWSESGNKQASGGNDNVVHIWD---RSLASSKQTRQWLHRFEEHTAAVRALAWCPFQAS 314

AT5G27080 LKWSESGKKLASGGNYNVVHIWDH--RSVASSKPTRQWLHRFEEHTAAVRALAWCPFQAT 312

AT5G27570 LKWSESGKKLASGGNDNVVHIWDH--RSVASSNPTRQWLHRFEEHTAAVRALAWCPFQAS 320

AL6G28380 LKWSESGKQLASGGNDKVVHIWD---RSLASSNSTRKWLQRFEGHTAATKALAWCPFQAN 310

AL7G07950 LKWSGSGQQLASGGNDNVVHIWD---RSVASSNSNTQWLHRLEEHTSAVKALAWCPFQAN 327

AT4G33270 LKWSGSGQQLASGGNDNVVHIWD---RSVASSNSTTQWLHRLEEHTSAVKALAWCPFQAN 328

AT4G33260 LKWSGSGQQLASGGNDNVVHIWD---RSVASSNSTTQWLHRLEEHTSAVKALAWCPFQAN 318

PT13G04450 LKWSASGQQLASGGNDNLIHIWDR--STALSNS-ATQWLHRLEDHTSAVKALAWCPFQGN 326

PT19G03660 LKWSASGQQLASGGNDNLIHIWDR--STALSNS-ATQWLHRLEDHTSAVKALAWCPFQGN 321

PT16G11830 LKWSASGQQLASGGNDNIIHIWDR--SVASSNS-ATQWFHRLEEHTSAVKALAWCPFQGN 331

CP00039G01100 LKWSGSGQKLASGGNDNLVHIWDR--SLASSSSERQQWLHRLEEHTSAVKALAWCPFQGN 334

CP01199G00020 LKWSGSGQQLASGGNDNLLHIWDR--SMASSNS-ATQWLHRLEEHTSAVKALAWCPFQGN 336

GM01G43980 LKWSASGSQLASGGNDNLLYIWD---RATASSNSATQWLHRLEDHTSAVKALAWCPFQGN 326

GM11G01450 LKWSASGSQLASGGNDNLLYIWD---RATASSNSATQWLHRLEDHTSAVKALAWCPFQGN 326

VV15G00180 LKWSASGQQLASGGNDNLLHIWD---RSSASSNSPTQWLHRMEDHTAAVKALAWCPFQGN 321

GM03G36300 LRWSPSGQQLASGGNDNVIHIWD---RTMVSSNSPTHWLHRFEEHRAAVKALAWCPFQAN 329

GM08G24480 LRWSPSGQQLASGGNDNVIHIWD---RAMVSSNSPTRWLHRFEEHKAAVRALAWCPFQAN 329

VV05G00820 LKWSTSGQQLASGGNDNLLYIWD---RSMASMHSRSQWLHRLEDHTAAVKALAWCPFQRN 315

VV05G00850 LKWSTSGQQLASGGNDNLLHIWD---RSMASMHSRSQWLHRLEDHTAAVKALAWCPFQRN 315

VV05G00770 LKWSTSGQQLASGGNDNLLYIWD---RSMASMHSRSQWLHRLEDHTAAVKALAWCPFQRN 315

VV05G06570 LKWSTSGQQLASGGNDNLLYIWD---RSMASMHSRSQWLHRLEDHTAAVKALAWCPFQRN 315

VV16G06260 LKWSASGQQLASGGNDNMLYIWD---RSMSSSNSRSQWLHRLEDHTAAVKALAWCPFQSN 325

SB04G009980 LKWSGSGQQLASGGNDNLLHIWDVSMASSMPSAGRNQWLHRLEDHTAAVKALAWCPFQSN 347

ZM05G20580 LKWSGSGQQLASGGNDNLLHIWDVSMASPMSTAGRNQWLHRLEDHMSAVKALAWCPFQSN 342

OS02G47180 LKWSGSGQQLASGGNDNLLHIWDVSMASSVPSAGRNQWLHRLEDHTAAVKALAWCPFQSN 340

ZM04G17500 LKWSGSGQQLASGGNDNLLHIWDVSMASSMPSAGRNQWLHRLEDHMAAVKALAWCPFQSN 348

OS04G51110 LKWSGSGQQLASGGNDNLLHIWDVSMASSMPSAGRTQWLHRLEDHLAAVKALAWCPFQSN 343

PT16G06730 LKWSASGQQLASGGNDNLLFIWD---RFMASSNSPRHWLHKLEDHTAAVKALAWCPFQSN 320

*:** **.: ***** ::: :*: : ::::: * :*.:********

AL4G08930 LLATGGGGEDRTIKFWNTRTGACLNSVDTGSQVCSLIWSNKERELLSSHGFTQNQLTLWK 383

AL5G12940 LLATGGGGEDRTIKFWNTRTGACLNSVDTGSQVCSLIWSKKERELLSSHGFTQNQLTLWK 383

AT5G26900 LLATGGGVGDGKIKFWNTHTGACLNSVETGSQVCSLLWSQSERELLSSHGFTQNQLTLWK 374

AT5G27080 LLATGGGVGDGKIKFWNTHTGACLNSVETGSQVCSLLWSQRERELLSSHGFTQNQLTLWK 372

AT5G27570 LLATGGGVGDGKIKFWNTHTGACLNSVETGSQVCSLLWSKSERELLSSHGFTQNQLTLWK 380

AL6G28380 LLATGGGVGDRTIKFWNTHTGACLNSVETGSQVCSLLWSNKERELLSSHGFTQNQLTLWK 370

AL7G07950 LLATGGGGGDRTIKFWNTHTGACLNSVDTGSQVCSLLWSKNERELLSSHGFTQNQLTLWK 387

AT4G33270 LLATGGGGGDRTIKFWNTHTGACLNSVDTGSQVCSLLWSKNERELLSSHGFTQNQLTLWK 388

AT4G33260 LLATGGGGGDRTIKFWNTHTGACLNSVDTGSQVCSLLWSKNERELLSSHGFTQNQLTLWK 378

PT13G04450 LLASGGGGGDKSIKFWNTHTGACLNSIDTGSQVCSLLWNKNERELLSSHGFTQNQLTVWK 386

PT19G03660 LLASGGGGGDKSIKFWNTHTGACLNSIDTGSQVCSLLWNKNERELLSSHGFTQNQLTVWK 381

PT16G11830 LLASGGGGGDRSIKFWNTHTGACLNSIDTGSQVCALLWNKNERELLSSHGFTQNQLVLWK 391

CP00039G01100 LLASGGGEGDRCIRFWNTQTAACLNTVDTGSQVSSLLWNKKERELLSSHGFFHNQLTLWK 394

CP01199G00020 LLASGGGGGDRCIKFWNTHTGACLNTVDTGSQVCALLWNKNERELLSSHGFTHNQLTLWK 396

GM01G43980 LLASGGGSGDRCIKFWNTHTGACLNSIDTGSQVCSLLWNKNERELLSSHGFTQNQLTLWK 386

GM11G01450 LLASGGGSGDRCIKFWNTHTGACLNSIDTGSQVCSLLWNKNERELLSSHGFTQNQLTLWK 386

VV15G00180 LLASGGGGGDRCIKFWNTHTGACLNSVDTGSQVCALLWNKNERELLSSHGFTQNQLTLWK 381

GM03G36300 LLASGGGGGDHCIKFWNTHTGACLNSVDTGSQVCALLWSKNERELLSSHGFTQNQLALWK 389

GM08G24480 LLASGGGGGDHCIKFWNTHTGACLNSVDTGSQVCALVWNKNERELLSSHGFTQNQLALWK 389

VV05G00820 LLASGGGGSDGCIKFWNTHTSACLNSVDTGSQVCALLWNKNERELLSSHGFMQNQMTLWM 375

VV05G00850 LLASGGGGSDGCIKFWNTHTGACLNSVDTGSQVCALLWNKNERELLSSHGFMQNQMTLWM 375

VV05G00770 LLASGGGGSDSCIKFWNTHTGACLNSVDTGSQVCALLWNKNERELLSSHGFMQNQMTLWM 375

VV05G06570 LLASGGGGSDCCIKFWNTHTGACLNSVDTGSQVCALLWNKNERELLSSHGFMQNQLTLWM 375

VV16G06260 LLASGGGGNDLCIRFWNTHTGACLNTVDTGSQVCALLWNKKERELLSSHGFSQNQLTLWK 385

SB04G009980 LLATGGGGSDRCIKFWNTHTGACLNSVDTGSQVCALLWNKNERELLSSHGFTQNQLTLWK 407

ZM05G20580 LLATGGGGSDRCIKFWNTHTGACLNSVNTGSQVCALLWNKNERELLSSHGFTQNQLTLWK 402

OS02G47180 LLATGGGGSDRCIKFWNTHTGACLNSVDTGSQVCALLWNKNERELLSSHGFTQNQLTLWK 400

ZM04G17500 LLATGGGGSDRCIKFWNTHTGVCLNSVDTGSQVCALLWNKNERELLSSHGFTQNQLTLWK 408

OS04G51110 LLASGGGGSDRCIKFWNTHTGACLNSIDTGSQVCSLVWNKNERELLSSHGFAQNQLTLWK 403

PT16G06730 LLASGGGGNDRHIKFWNTQTGTCLNSVDTGSQVCALQWNKHERELLSSHGFTENQLILWK 380

***:*** * *:****:*..***:::*****.:* *.: ********** .**: :*

AL4G08930 YPSMVKMAELNGHTSRVLYMSQSPDGCTVASAAGDETLRLWNVFGIPE--DAKKAAPKA- 440

AL5G12940 YPSMVKMAELNGHTSRVLYMSQSPDGCTVASAAGDETLRLWNVFGVPE--DAKKAAPKA- 440

AT5G26900 YPSMSKMAELNGHTSRVLFMAQSPNGCTVASAAGDENLRLWNVFGEPP-KTTKKAASKK- 432

AT5G27080 YPSMSKMAELNGHTSRVLFMAQSPNGCTVASAAGDENLRLWNVFGEPP-KTTKKAASKN- 430

AT5G27570 YPSMVKMAELNGHTSRVLFMAQSPDGCTVASAAGDETLRLWNVFGEPP-KTTKKAASKK- 438

AL6G28380 YPSMLKIAELNGHTSRVLYMAQSPDGCTVASAAGDETLRLWNVFGVPPPKTTKKAAPKA- 429

AL7G07950 YPSMVKMAELTGHTSRVLYMAQSPDGCTVASAAGDETLRFWNVFGVPE--TAKKAAPKA- 444

AT4G33270 YPSMVKMAELTGHTSRVLYMAQSPDGCTVASAAGDETLRFWNVFGVPE--TAKKAAPKA- 445

AT4G33260 YPSMVKMAELTGHTSRVLYMAQSPDGCTVASAAGDETLRFWNVFGVPE--TAKKAAPKA- 435

PT13G04450 YPSMVKMAELTGHTSRVLYMAQSPDGCTVATAAGDETLRFWNVFGVPE--VAAKAAPKA- 443

PT19G03660 YPSMVKMAELTGHTSRVLYMAQSPDGCTVATAAGDETLRFWNVFGVPE--IAAKAAPKA- 438

PT16G11830 YPSMLKMAELTGHTSRVLYMAQSPDGCTVATAAGDETLRFWNVFGVPE--VA-KAAPKA- 447

CP00039G01100 YPSMMKVAELTGHKSRVLCMAQSPDGCTVASAAGDERVKLWNVFGVPE--KAAKAARKQ- 451

CP01199G00020 YPSMVKIAELTGHTSRVLYMAQSPDGCTVASAAGDETLRFWNVFGVPE--TAAKAAPKQ- 453

GM01G43980 YPSMVKMAELTGHTSRVLFMAQSPDGCTVASAAADETLRFWNVFGAPE--AASKAAPKA- 443

GM11G01450 YPSMVKMAELNGHTSRVLFMAQSPDGCTVASAAADETLRFWNVFGAPE--AASKAAPKA- 443

VV15G00180 YPSMVKMAELTGHTSRVLFMAQSPDGCTVASAAGDETLRFWNVFGTPE--VAAKPAPKA- 438

GM03G36300 YPSMLKMAELKGHTSRVLYMAQSPNGCTVASAAGDETLRFWNVFGTAQ---ASKPAPTA- 445

GM08G24480 YPSMLKKAELKGHTSRVLYMAQSPNGCTVASAAGDETLRFWNVFGTPQ---ASKPAPKT- 445

VV05G00820 YPSMVKIAELTGHTSRVLFMAQSPDGRTVATAAGDETLKFWNAFG-TP--EVKKAAPKAE 432

VV05G00850 YPSMVKIAELTGHTSRVLFMAQSPDGRIVATAAGDETLKFWNAFG-TP--EVKKAAPKAE 432

VV05G00770 YPSMVKIAELTGHTSRVLFMAQSPDGRTVATAAGDETLKFWNAFG-TP--EVKKASPKAE 432

VV05G06570 YPSMVKTAELTGHTSRVLFMAQSPDGRTVATAAGDETLKFWNAFG-MP--EVKKAAPKAE 432

VV16G06260 YPSMVKITELTGHTSRVLFMAQSPDGCTVVTAAGDETLKFWNVFGTTP--EVKNAAPK-- 441

SB04G009980 YPSMVKMAELTGHTSRVLFMAQSPDGCTVASAAADETLRFWNVFGAPE---APKP-VKAS 463

ZM05G20580 YPSMVKMAELSGHTSRVLFMAQSPDGCTVASAAADETLRFWNVFGDPE---VAKPAAKAS 459

OS02G47180 YPSMVKMAELTGHTSRVLFMAQSPDGCTVASAAADETLRFWNVFGSPE---APKPAAKAS 457

ZM04G17500 YPSMVKMAELNGHTSRVLFMAQSPDGCTVASAAADETLRFWNVFGTPE---TPKPAAKAS 465

OS04G51110 YPSMVKMAELTGHTSRVLFTAQSPDGLTVASAAADETLRFWNVFGAPE---APKTATKGS 460

PT16G06730 YPSMVKMAELSGHTSPVLFMTQSPDGYTVASAAGDETLRFWNVFGNPK---AAKPAPKA- 436

**** * :**.**.* ** :***:* *.:**.** :::**.** . :. .

AL4G08930 VPQPFSNVN--RIR 452

AL5G12940 VPQPFSNVN--RIR 452

AT5G26900 YPELFSHVN--SLR 444

AT5G27080 YLELFSHVN--SLR 442

AT5G27570 YTDPFAHVN--HIR 450

AL6G28380 YLQIFSHVN--CIR 441

AL7G07950 VSEPFSHVN--RIR 456

AT4G33270 VSEPFSHVN--RIR 457

AT4G33260 VAEPFSHVN--RIR 447

PT13G04450 NPEPFSHLN--RLR 455

PT19G03660 NPEPFSHLN--RIR 450

PT16G11830 NPEPFSRFN--RIR 459

CP00039G01100 NREPFSHLS--RIR 463

CP01199G00020 NPEPFSHLN--RIR 465

GM01G43980 RAEPFSNVN--RIR 455

GM11G01450 RAEPFSNVN--RIR 455

VV15G00180 HPEPFAHLN--RIR 450

GM03G36300 STDPFAHVN--RIR 457

GM08G24480 NVEPFANVN--CIR 457

VV05G00820 HPGPFPHLR--RIR 444

VV05G00850 HPGPFPHLR--RIR 444

VV05G00770 HPGPFPHIR--RIR 444

VV05G06570 HPGPFPHIR--RIR 444

VV16G06260 --ELFPHFS--RIR 451

SB04G009980 HTGMFNSFN--HIR 475

ZM05G20580 HTGMFNSFN--HIR 471

OS02G47180 HTGMFNSFN--HLR 469

ZM04G17500 HTGMFNSFK--HIR 477

OS04G51110 HTGMFNNSNHIHIR 474

PT16G06730 IAEPFANVS--HFR 448

* ::
