# Supplementary figures and images for: Conserved CDC20 Cell Cycle Functions Are Carried out by Two of the Five Isoforms in Arabidopsis thaliana
Source: PLoS One. 2011 Jun 8;6(6):e20618. doi: 10.1371/journal.pone.0020618 (PMC3110789; doi:10.1371/journal.pone.0020618)

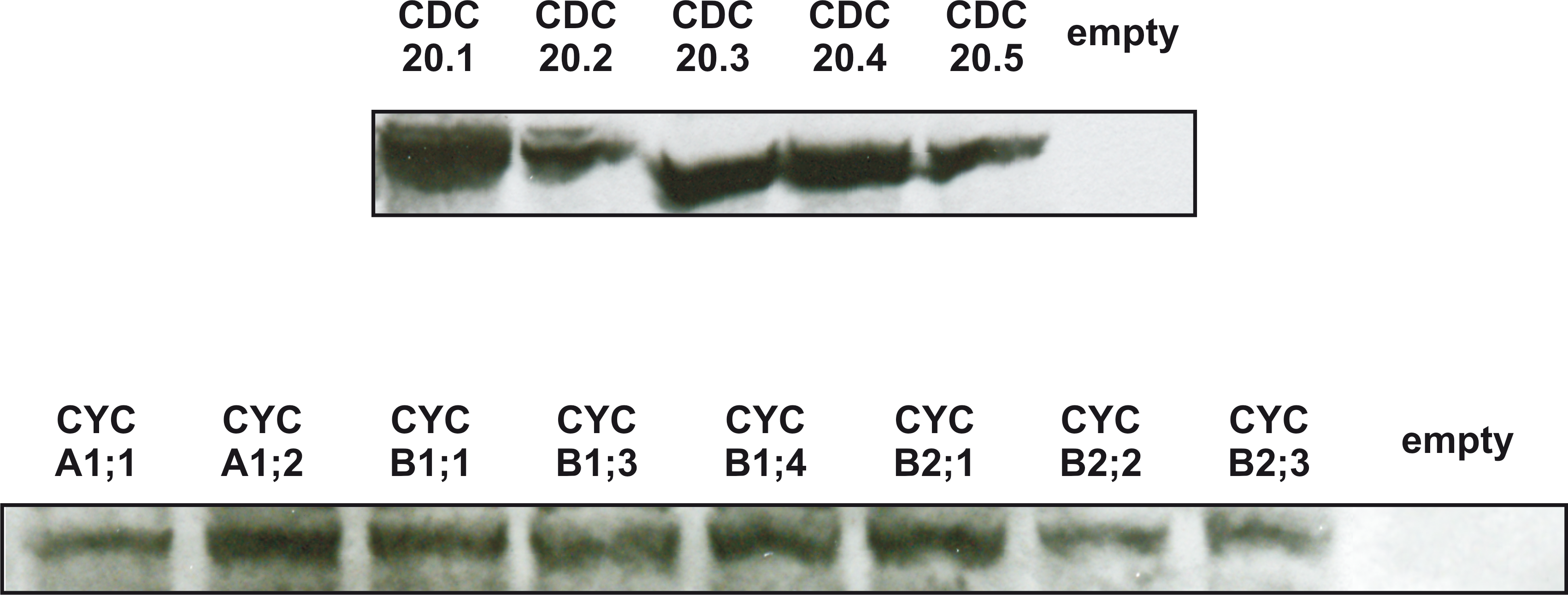

Supplement: Figure S4 — Production of the Arabidopsis CDC20 isoforms and mitotic cyclins in yeast cells. The presence of CDC20 and cyclin proteins expressed from the Y2H pGADT7 vector was detected in yeast total protein extracts by Western Blot analysis with the anti-HA antibody. Upper panel, production of the five AtCDC20 proteins as indicated. Lower panel, production of the Arabidopsis cyclin proteins as indicated. Empty corresponds to the analysis of protein extracts of yeast containing the empty pGADT7 vector. (TIF) [file pone.0020618.s004.tif]
